# Stratum Corneum Sampling to Assess Bioequivalence between Topical Acyclovir Products

**DOI:** 10.1007/s11095-019-2707-3

**Published:** 2019-11-14

**Authors:** A. Pensado, W.S. Chiu, S. F. Cordery, E. Rantou, A. L. Bunge, M. B. Delgado-Charro, R. H. Guy

**Affiliations:** 1 Department of Pharmacy & Pharmacology, University of Bath, Claverton Down, Bath, BA2 7AY UK; 2 Office of Biostatistics, Office of Translational Sciences, Center for Drug Evaluation and Research, United States Food and Drug Administration, White Oak Campus, Silver Spring, MD USA; 3 Department of Chemical and Biological Engineering, Colorado School of Mines, Golden, CO USA

**Keywords:** acyclovir, skin, topical bioavailability, topical bioequivalence, stratum corneum sampling, scaled average bioequivalence (SABE)

## Abstract

**Purpose:**

To examine the potential of stratum corneum (SC) sampling via tape-stripping in humans to assess bioequivalence of topical acyclovir drug products, and to explore the potential value of alternative metrics of local skin bioavailability calculable from SC sampling experiments.

**Methods:**

Three acyclovir creams were considered in two separate studies in which drug amounts in the SC after uptake and clearance periods were measured and used to assess bioequivalence. In each study, a “reference” formulation (evaluated twice) was compared to the “test” in 10 subjects. Each application site was replicated to achieve greater statistical power with fewer volunteers.

**Results:**

SC sampling revealed similarities and differences between products consistent with results from other surrogate bioequivalence measures, including dermal open-flow microperfusion experiments. Further analysis of the tape-stripping data permitted acyclovir flux into the viable skin to be deduced and drug concentration in that ‘compartment’ to be estimated.

**Conclusions:**

Acyclovir quantities determined in the SC, following a single-time point uptake and clearance protocol, can be judiciously used both to objectively compare product performance in vivo and to assess delivery of the active into skin tissue below the barrier, thereby permitting local concentrations at or near to the site of action to be determined.

**Electronic supplementary material:**

The online version of this article (10.1007/s11095-019-2707-3) contains supplementary material, which is available to authorized users.

## Introduction

Topical drug products containing acyclovir (ACV) are indicated for the treatment of recurrent cutaneous herpes labialis (cold sores) in immunocompetent adults and adolescents 12 years of age and older, a common infective skin condition primarily caused by herpes simplex virus type 1 (HSV-1) (1). HSV-1 infections occur in the basal epidermis (2) meaning that the effectiveness of acyclovir against the virus depends on drug release and penetration through the stratum corneum (SC) to reach this target. Indeed, this drug delivery challenge was recognised early on in the use of antiviral compounds and the idea was proposed that early application of topical therapy should, in principle, be able to alter the course of cold sore development, and even to prevent lesion outbreak (3).

Evidence of topical ACV efficacy for the treatment of cold sores was established in clinical trials in the 1980s (4–6), and was later confirmed in two independent, randomized, double-blind, vehicle-controlled clinical trials that demonstrated a statistically significant reduction in the duration of lesion pain (7). An additional, similar placebo (vehicle)-controlled clinical trial in patients with recurrent herpes labialis showed that the mean duration of the viral episode was approximately half a day shorter after treatment with the reference listed drug (RLD) product in the US, Zovirax® (acyclovir) cream, 5%, as compared with subjects treated with a placebo (vehicle alone) control (8); however, no significant difference was observed between treated and control patients with respect to the progression of cold sore lesions (8).

Presently, in the US, there is no generic ACV cream approved by the Food and Drug Administration (FDA). In fact, with the exception of certain topical solutions, the corticosteroids, and a handful of other topical drug products, which are the subject of recently released product-specific guidances (9), a clinical trial has been the default approach for the approval of a generic product. This has been a recognised barrier to the entry of such formulations into the market because the comparative clinical trials required are often poorly discriminating between products and are time and resource expensive as a result, because large numbers of patients are needed to ensure sufficient statistical power (10). Hence, for a generic ACV cream, 5%, to have been approved, it would have to be bioequivalent to Zovirax® and would need to show, in accord with the FDA definition, and relative to the RLD: “*the absence of a significant difference in the rate and extent to which the active ingredient or active moiety in pharmaceutical equivalents or pharmaceutical alternatives becomes available at the site of drug action when administered at the same molar dose under similar conditions in an appropriately designed study”* (11).

The requirement for a clinical endpoint bioequivalence study reflects the challenge confronted rather generally by locally acting drugs (including topical drug products applied to the skin), the site of action of which is not attained by absorption into the systemic blood and subsequent distribution. As a result, there is an ongoing and intensifying effort to identify and validate surrogate methods for the assessment of topical bioequivalence. Recent work has focussed upon (a) in vivo microdialysis (and, specifically, open-flow microperfusion) (12), (b) in vivo SC sampling (i.e., tape-stripping) (13,14), and (c) in vitro permeation testing (IVPT) using excised human skin mounted on diffusion cells (e.g., Franz cells) (13,15).

In fact, a concerted investigation has been launched to compare the RLD with other 5% w/w ACV creams approved for use outside of the US using each of the alternative methods for evaluating bioequivalence. One component study involving open-flow microperfusion has already been published and reported the conclusion that the bioavailability of ACV from an Austrian generic product (Aciclovir A1 Pharma) was lower than that from the RLD (12). The results of IVPT experiments from three laboratories (two in the US, one in Australia) – again, comparing the RLD with other ACV creams – are published (16) concurrently with research described in this paper that has used in vivo SC sampling to assess the performance of the U.K. version of Zovirax®, and of the same Austrian generic mentioned above, with that of the RLD.

The SC sampling approach employed has followed closely the improved protocol reported in 2009 (17) that was designed to overcome a number of limitations of the method specified in a FDA draft guidance (18) first published in 1999. This guidance was withdrawn in 2002 (19) following inconsistency in the results reported from two expert laboratories when using the same tape-stripping protocol to compare the bioequivalence of two generic tretinoin gel formulations to that of the reference-listed product (20,21). In contrast, the improved protocol (17) has been shown to be robust in its performance, having accurately reflected the clinical bioequivalence of two econazole nitrate creams to the innovator product (17) and successfully distinguished a clearly different diclofenac product from two other formulations of the same drug (14). Not only were these latter results consistent in a qualitative way with parallel IVPT measurements, the deduced drug fluxes into the underlying viable skin in vivo were also quantitatively very similar to those measured in vitro.

There is support, therefore, for the hypothesis that the SC sampling method based on a simplified, but rigorous, tape-stripping protocol is, first of all – and, perhaps, unsurprisingly – a credible ‘reporter’ of the topical bioavailability of drugs, whose site of action is on and/or within the SC, and that, in addition, can also provide useful metrics related to the bioavailability of active moieties, which act either within the viable skin layers (i.e., beyond the SC) or even below them. With this approach, SC sampling is undertaken at two distinct moments in time: first, after an ‘uptake’ period, during which a concentration gradient of the drug is achieved across the barrier and then, second, following a further period in which the concentration profile in the SC is allowed to dissipate as the drug is ‘cleared’ into the underlying viable skin (17). In the present work, the enhanced SC sampling protocol is extended to the comparison of three ACV creams to evaluate topical bioavailability and bioequivalence and, furthermore, to demonstrate the potential of the approach to assess whether a target concentration at the putative site of drug action (in this case, the basal epidermis) has been achieved.

## Materials and Methods

### Materials

The study examined three commercially available acyclovir (ACV) creams, Zovirax® (Valeant Pharmaceuticals, Bridgewater, US) (ACV-US), Zovirax® (GlaxoSmithKline Consumer Healthcare, Brentford, UK) (ACV-UK), and Aciclovir 1A Pharma Cream (1A Pharma GmbH, Vienna, Austria) (ACV-AT) that contain 5% acyclovir (50 mg ACV per gram of product) but differ in the type and/or amount of the inactive ingredients (Table I). Pure ACV was obtained from Sequoia Research Products, Ltd. (Pangbourne, UK); solvents and HPLC reagents were from Sigma Aldrich (Gillingham, UK).

**Table I:** Components of the 5% (*w*/*w*) Acyclovir Products Tested (all Dispensed from Tubes)

Product		Excipients
Zovirax® (US)	(ACV-US)	Cetostearyl alcohol, mineral oil, poloxamer 407, propylene glycol, sodium lauryl sulfate, water and white petrolatum.
Zovirax® (UK)	(ACV-UK)	Dimeticone, propylene glycol, poloxamer 407, cetostearyl alcohol, sodium lauryl sulfate, white soft paraffin, liquid paraffin, arlacel 165 (glycerol monosterate, macrogol stearate 100) and purified water.
Aciclovir 1A Pharma (Austria)	(ACV-AT)	Glycerol monosterate, polyoxyethylenstearate, dimeticone, cetylacohol, white soft paraffin, liquid paraffin, propylene glycol, purified water.

### Design of the Investigation

The investigation was designed in two parts. Study 1 compared ACV-US (US-Ref) with ACV-UK (UK-Test), with the former tested twice to provide a positive control (US-C+). Study 2 compared ACV-US (US-Test) and ACV-AT, with the latter tested twice in this case (AT-Ref and AT-C+). The protocol was approved by both the Research Ethics Approval Committee for Health at the University of Bath, and the FDA’s Research Involving Human Subjects Committee. Informed consent was obtained from each subject. Ten healthy volunteers, without a history of dermatological disease and with healthy skin on the volar surface of both arms, were enrolled in each study. In Study 1, 7 females and 3 males (9 white and 1 Asian, age range 24–52 years) participated in the study; in Study 2, there were 5 females and 5 males (6 white, 1 black and 3 Asian, age range 23–29 years). There was no overlap of the participants between the two studies. For subjects with high hair density, the ventral forearms were shaved using a new disposable razor at least 24 h before the study began. No lotion, cream or other personal care product was used on the forearms for at least 24 h before and during the study.

#### Drug Application

The procedures adopted followed closely the method developed by N’Dri-Stempfer et al. (17) to overcome the shortcomings of the original FDA tape-stripping guidance (18). Briefly, the approach assesses the amount of drug in the SC (a) after a specific ‘uptake’ period following application of the formulation (which was 6 h in both studies), and (b) following a defined ‘clearance’ period (17 h in this work) after removal of the product at the end of ‘uptake’. For each product considered, duplicate measurements of ‘uptake’ and ‘clearance’ are made. Therefore, in both Study 1 and Study 2 (each of which compared three treatments), 12 application sites, 6 per forearm, were required.

**Fig. 1:**
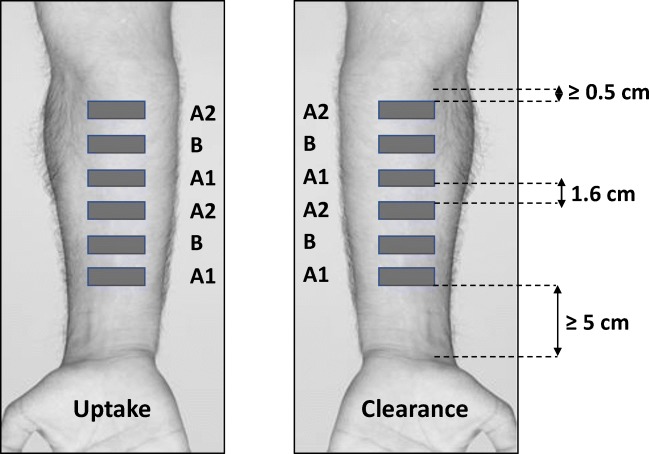
Schematic diagram illustrating the randomised distribution of product application sites (2 × 2 duplicates each of product A and 1 × 2 duplicates of product B). Note: dimensions are not to scale.

The treatment sites were demarcated using rectangular-shaped frames with an 8.25 cm^2^ (1.5 cm × 5.5 cm) open area cut from self-stick adhesive (Pressure Point Foam Padding, Scholl, Slough, UK). The sites were separated by 1.6 cm and located at least 5 cm above the wrist and a minimum of 0.5 cm below the antecubital fossa (Fig. 1). Each volunteer was asked to select one arm for the ‘uptake’ measurements, the other being used for ‘clearance’. On the upper half of the ‘uptake’ arm, the three application sites of the three products were randomly assigned, and this order was duplicated on the lower part of the arm; the same randomised order was mirrored on the other arm for the clearance measurements (Fig. 1).

One hour before drug was applied, the skin was cleaned with a standard soap and water wash (Carex Complete, Cussons, Manchester, UK). The nominal “dose” of each cream (15 mg/cm^2^) was applied to the skin site using a cotton bud (Johnson & Johnson, Berkshire, UK) to spread and massage the product into the demarcated area. The exact loading of the formulation applied was determined by subtracting the weight of residual product on the earbud from the quantity of product applied. Immediately post-application, the treated sites were protected with a non-occlusive plastic mesh (Ultra stiff plastic canvas, 7 mesh, Darice®, OH, US) held on - but without touching - the skin by Mefix® tape (Molnlycke, Lancashire, UK). At the end of the 6-h ‘uptake’ period, the protective mesh and the frame were removed, and residual drug was cleaned from all the treated skin sites, first with one dry wipe (Wypall, Kimberly Clark, Kent, UK) and then with two 70% isopropyl alcohol wipes (Sterets®, Molnlycke, Lancashire, UK).

#### SC Sampling

The use of tape-stripping to sample the SC has been fully described in the literature (13,14,17). All sites on the designated uptake forearm were tape-stripped immediately after drug removal. The edges of the treatment areas on the clearance arm were demarcated using Mefix® tape (Molnlycke, Lancashire, UK), without encroaching on the treated area. The whole forearm was covered with light gauze (Boots, Nottingham, UK) to protect the area overnight. Seventeen hours later, all sites on the clearance forearm were tape-stripped.

Immediately prior to tape-stripping, a thin template of Scotch® Book Tape (3 M, St. Paul, MN, US) was used to define a central 5 cm^2^ area (1 cm × 5 cm) of the drug application site. All sites were tape-stripped by the repeated application of adhesive (Scotch® Book Tape) tape-strips (1.5 × 6.5 cm) that overlapped the edges of the template. Each tape was pressed firmly to the skin, with rubbing for a few seconds, and then removed in alternating directions for successive strips. To ensure that most of the SC was removed, without complete derangement of the barrier, transepidermal water loss (TEWL) was measured (AquaFlux® evaporimeter, Biox System Ltd. London, UK) before and during the tape-stripping process (22,23). Tape-stripping was stopped if any one of the following occurred: (a) TEWL reached 60 g·m^−2^·h^−1^, (b) the TEWL value exceeded 6 times the baseline pre-stripping value, or (c) 30 tapes had been removed.

The mass of SC removed was determined by weighing the tapes (Microbalance SE-2F, precision 0.1 μg; Sartorius AG, Göttingen, Germany) before and after tape-stripping; to ensure accurate measurements, tapes were discharged of static electricity (R50 discharging bar and ES50 power supply Eltex Elektrostatik GmbH, Germany) before being weighed.

#### Drug Extraction and Analysis

Drug was extracted from groups of tape-strips into 3.6 mL of 30:70 methanol:water by sonication for 1 h followed by shaking overnight at room temperature. Samples were filtered (0.45 μm nylon membrane, SMI-Labhut, Ltd., Maisemore, UK) and transferred to HPLC vials for analysis.

The tape-strips were grouped to increase the likelihood that the aggregated samples contained a sufficient drug amount to exceed the limit of quantification of the assay (see below). Typically, the first two tape-strips were extracted separately, while the remainder were groups of 2 to 8 tapes determined primarily by approximately equalising the weights of SC removed.

The extracted ACV was quantified by HPLC (Shimadzu LC-2010, Buckinghamshire, UK) with UV detection (254 nm). A mobile phase of 17.5:82.5 methanol:0.1% acetic acid was pumped at a flow rate of 1 mL/min through a 250 × 4.6 mm HiQ Sil C18 column (Kromatek, Dunmow, UK). The injection volume was 50 μL and the retention time of ACV was ~5 min; limits of quantification and detection were 0.038 and 0.01 μg/mL, respectively. Calibration standards were measured in triplicate while all tape-strip samples were analysed in duplicate. The amount of drug per unit area in the SC (Q) was calculated as the sum of drug mass in the extracts of all tape groups from each site normalized by the sample area. The measured extract concentrations were greater than the LOQ in all tape strip groups except from one site in one volunteer (subject 3 treated with UK-Test at clearance), for which Q was assigned the value of LOQ/2 (i.e., 0.0137 μg/cm^2^). The justification for this assumption was the subsequent requirement in the statistical analysis of the results to log-transform the Q values, an impossibility, of course, when Q = 0.

Control samples of SC that had not been exposed to any ACV-containing formulation were acquired from each volunteer and subjected to the identical extraction and analysis procedures to confirm the absence of any interference in the chromatogram at the retention time of the drug.

### Data Analysis

The thickness of the SC removed by tape-stripping was calculated from the mass of SC on each tape divided by the area sampled and the density of the SC, assumed to be 1 g/cm^3^ (24). The arithmetic means of the total SC mass collected in the duplicate sites were calculated and then averaged across the 10 subjects for ‘uptake’ and ‘clearance’ of each product. The mass of drug in the SC was expected to exhibit a log-normal distribution (25–27). Therefore, the arithmetic average, standard deviation and 90% confidence intervals of the logarithm of the geometric mean of the duplicate measurements in the 10 subjects were calculated for ‘uptake’ and ‘clearance’ of each product (17).

The average flux of drug transferred from the SC to the underlying tissue (J_in vivo_) during ‘clearance’ was calculated from the geometric mean of the drug mass in the duplicated sites for each product in each subject as:

1 $$ {\mathrm{J}}_{\mathrm{in}\ \mathrm{vivo}}=\left({\mathrm{Q}}_{\mathrm{Up}}-{\mathrm{Q}}_{\mathrm{Cl}}\right)/\Delta \mathrm{t} $$

where Q_Up_ is the mass per unit area of drug in the SC at the end of the 6-h period of ‘uptake’, Q_Cl_ is mass per unit area of drug in the SC 17 h after removal of the residual formulation, and Δt is the elapsed time between the ‘uptake’ and ‘clearance’ measurements, i.e., 17 h. Assuming that ACV is cleared from the SC with first-order kinetics, then the associated rate constant, k, is:

2 $$ \mathrm{k}=-\ln \left({\mathrm{Q}}_{\mathrm{Cl}}/{\mathrm{Q}}_{\mathrm{Up}}\right)/\Delta \mathrm{t} $$

Statistically significant differences were estimated by a two-tail t-test or by one-way ANOVA followed by Tukey’s test, assessed in a pairwise comparison within-subject where appropriate. In all the comparisons undertaken, statistical significance was set at *p* < 0.05. Reported 90% confidence intervals were calculated using the Student’s T-distribution for the sample size and the sample standard deviation.

#### Bioequivalence Evaluation

Bioequivalence of the products in the two studies was evaluated using the geometric mean of the duplicate values of the drug amount in the SC measured after 6 h of ‘uptake’ and 17 h of ‘clearance’ in each subject for each product as previously described (17). Applying the traditional average bioequivalence approach (28), the 90% confidence interval for the mean of the within-subject difference of the log-transformed drug amounts in the SC after ‘uptake’ or ‘clearance’ was calculated for the compared pair of products. Bioequivalence is established when the anti-log of the calculated confidence interval falls within the bioequivalence limit, traditionally 80–125% for the ratio of the population geometric means (29). Bioequivalence evaluation using ratios (i.e., the difference of log-transformed values) is inappropriate for comparing J_in vivo_ and k values from different products because data variability can cause negative values in these metrics. Therefore, a paired comparative assessment was performed by calculating the average and standard deviation of the differences of values between the products in each subject and testing the hypothesis that the true difference was zero against the alternative that it could be greater or less than zero. Example calculations of the bioequivalence evaluations for drug amounts in the SC, J_in vivo_ and k are provided in the Supplementary Materials for the comparison of the US-C+ and US-Test products (Tables S1 and S2).

## Results

The number of tapes collected and the mass of SC removed, at both uptake and clearance times in Study 1 and Study 2, and for each cream, are shown in Supplementary Fig. S1. The amount of SC removed may be affected by both intrinsic factors, such as anatomical site, as well as extrinsic factors, such as the adhesive tape used (30). That said, the effects of these factors on the measured drug mass are minimized as long as at least half of the SC is collected. Specifically, the outermost half of the SC will contain 75% of the total drug mass if the drug concentration profile has reached steady-state (i.e., it is linear with position in the SC), and an even larger fraction if steady-state has not been established and the concentration profile is not linear.

In this work, the average thicknesses of the SC collected in Studies 1 and 2, respectively, were 9.3 and 8.4 μm (corresponding to 0.84–0.93 mg of SC/cm^2^); that is, more than half the SC based on the reported total thickness on the ventral forearm of 10.9 ± 3.5 μm (23). No statistically significant differences were found in the number of tape-strips taken between the creams in Study 1 after uptake or in Study 2 after clearance; however, the number of tapes used after clearance for US-Ref and UK-Test in Study 1, and after uptake in Study 2 for US-Test and both AT-Ref and AT-C+ were significantly different. Within either Study 1 or Study 2, no statistically significant differences were found in the mass of SC removed between creams after uptake or clearance, except for the US-Test cream after uptake in Study 2, which was different from the AT-Ref and AT-C+ creams. Specifically, fewer tapes were necessary, and more SC was removed. This statistically significant difference is reflected in the average mass of SC collected per tape for the ACV-US cream compared with the ACV-AT creams during uptake (Study 2), which disappeared during clearance (Supplementary Fig. S2). Notably, this difference between uptake and clearance was not observed for the ACV-US creams in Study 1, perhaps because the variability in this case was greater. The more efficient tape-stripping of the Study 2 uptake sites treated with ACV-US may be due to the differences in excipient composition of the two formulations (e.g., the presence of sodium lauryl sulfate in ACV-US).

The ACV concentration plotted as a function of depth in the SC is presented in Supplementary Figs. S3 and S4 for Study 1 and Study 2, respectively. The concentration profiles in Study 1 for the 10 subjects at both uptake and clearance were similar for the three creams. However, in Study 2, the concentration profiles for the ACV-AT products were clearly different than that observed for the ACV-US cream after both uptake and clearance.

**Fig. 2:**
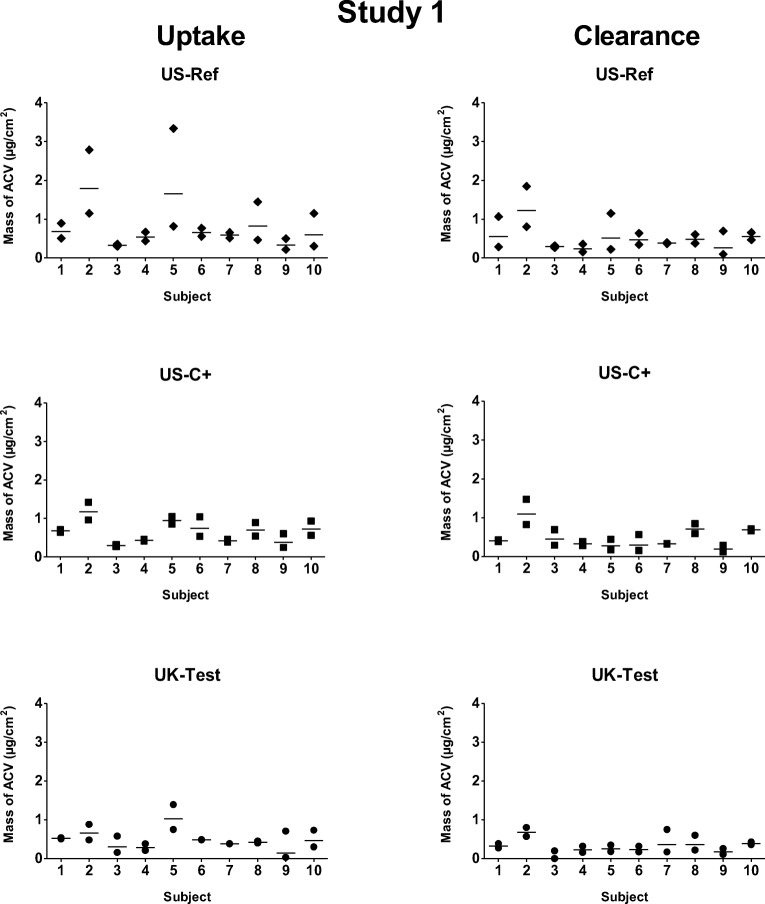
Mass of ACV (μg/cm^2^) recovered from the tape-strips in each subject from duplicate sites for each product (US-Ref, US-C+ and UK-Test) in Study 1 after the 6-h ‘uptake’ and 17-h ‘clearance’ periods. The line between symbols designates the geometric mean of the duplicate sites.

**Fig. 3:**
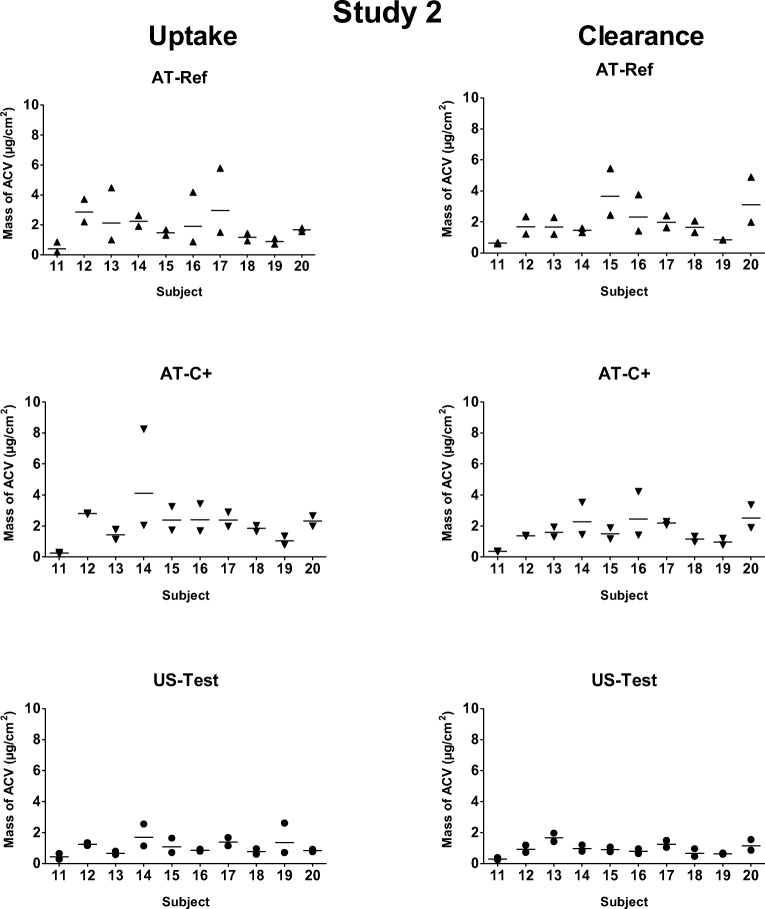
Mass of ACV (μg/cm^2^) recovered from the tape-strips in each subject from duplicate sites for each product (AT-Ref, AT-C+ and US-Test) in Study 2 after the 6-h ‘uptake’ and 17-h ‘clearance’ periods. Line between symbols designates the geometric mean of the duplicate sites.

**Fig. 4:**
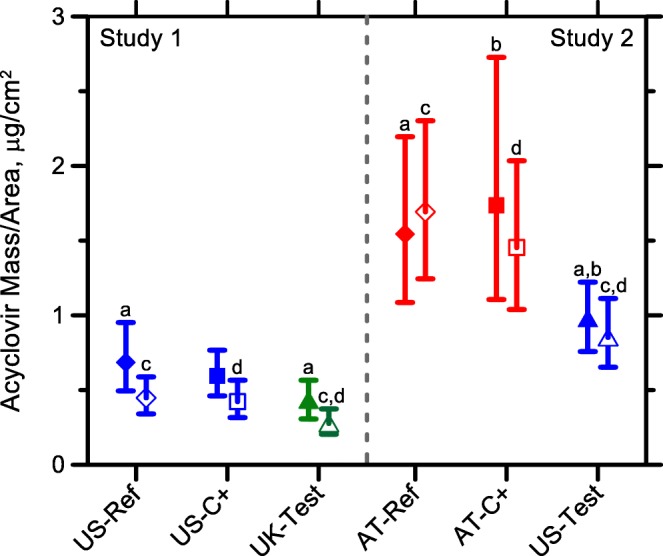
Mass of ACV in the SC (μg/cm^2^) for each product (mean and 90% confidence interval of the log-transformed average of the geometric mean of duplicates in each subject) after the 6-h ‘uptake’ (filled symbols) and 17-h ‘clearance’ (open symbols). Letters on pairs of creams indicate statistical difference in that study.

Figures 2 and 3 show the total amounts of ACV recovered from duplicate sites (for ‘uptake’ and ‘clearance’) of each subject in Study 1 and 2, respectively; Fig. 4 and Table II summarize these data from the 10 volunteers for each cream. In Study 1, at ‘uptake’, there was a statistically significant difference between the ACV masses recovered from the UK-Test and US-Ref sites, but no difference between UK-Test and US-C+. However, after ‘clearance’, there was a statistically significant difference between the drug amounts recovered from UK-Test and both US-Ref and US-C+ sites. At both ‘uptake’ and ‘clearance’ times of Study 1, no differences were found in the masses of drug recovered from the two ACV-US sites (US-Ref and US-C+). Similar behaviour was seen in Study 2: at both ‘uptake’ and ‘clearance’ time points, there was a statistically significant difference between the ACV masses recovered from US-Test and those from both AT-Ref and AT-C+ sites, but no difference between the AT-Ref and AT-C+ sites.

**Table II:** Average ACV Amounts Recovered from the SC After Uptake and Clearance (Q_Up_ and Q_Cl_, Respectively), the Deduced Drug Flux from the SC into the Underlying Viable Tissue During the Clearance Period (J_in vivo_), and the First-Order Rate Constant (k) Describing Clearance from the SC^a^

	Study 1				Study 2			
	US-Ref	US-C+	UK-Test	US-Ave	AT-Ref	AT-C+	US-Test	AT-Ave
Q_Up_ (μg/cm^2^)	0.68 (0.49–0.95)	0.59 (0.46–0.77)	0.42 (0.31–0.57)	0.64 (0.48–0.85)	1.54 (1.09–2.19)	1.74 (1.11–2.73)	0.96 (0.76–1.22)	1.64 (1.11–2.42)
Q_Cl_ (μg/cm^2^)	0.45 (0.34–0.59)	0.42 (0.32–0.57)	0.26 (0.17–0.38)	0.43 (0.33–0.56)	1.69 (1.24–2.30)	1.45 (1.04–2.03)	0.85 (0.65–1.11)	1.50 (1.24–2.13)
J_in vivo_ (ng/cm^2^/h)	18 ± 12	9.9 ± 8.2	9.6 ± 8.0	14 ± 8.6	−8.0 ± 37	27 ± 25	6.2 ± 17	9.6 ± 24
10^2^ k (h^−1^)	2.5 ± 1.2	2.0 ± 1.7	2.8 ± 2.2	2.3 ± 1.2	−0.5 ± 1.7	1.0 ± 1.2	0.7 ± 1.6	0.3 ± 1.2

^a^Q_Up_ and Q_Cl_ are reported as the anti-logarithm of the arithmetic mean (lower-upper 90% confidence interval) of the log-transformed values; J_in vivo_ and k are the arithmetic means (lower-upper 90% confidence intervals); *n* = 10. Values for US-Ave and AT-Ave were calculated using the geometric mean of 4 replicates (from Ref and C+ combined) for Q_up_ and Q_Cl_ in each subject

It is worth noting that, while the duplicate measurements made on each subject are generally close to one another, there are occasional exceptions such as subject 5, US-Ref (Study 1) and subject 14, AT-C+ (Study 2). As no experimental reason for these cases of high intra-subject/treatment variability was identifiable, these findings cannot be treated as formal outliers and must therefore be taken into account when defining the number of subjects required in a definitive bioequivalence assessment (an issue discussed further below and in the Supplementary Information). A similar comment is pertinent to the apparently inconsistent mean values of uptake and clearance amounts for AT-Ref in Study 2. In this case, the ability to discern a decrease in the amount of drug in the SC after clearance depends on the magnitude of the true change in drug amount relative to the variability of the measurement. The observation of larger drug amounts measured after clearance compared with uptake is not a surprise in an experiment with small changes in drug mass relative to the variability of the measurement of drug amount. This reinforces a previously emphasised point (17) that the ‘clearance’ time should be chosen so that it is long enough to increase the probability that a decrease in drug amount will be observed but not so long that drug amounts fall below the limits of detection.

## Discussion

**Fig. 5:**
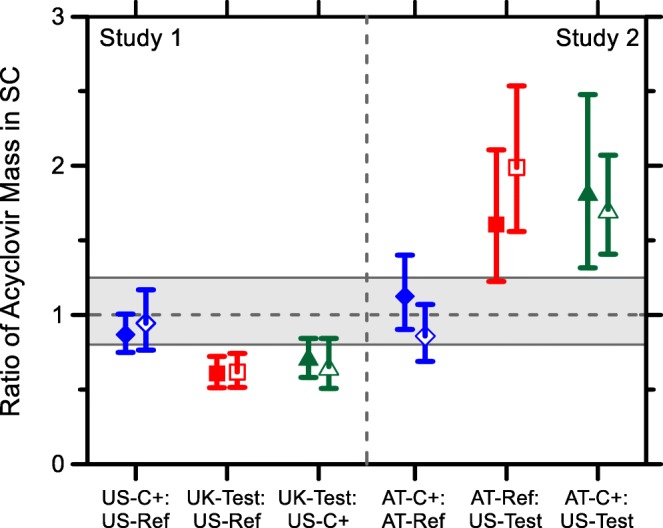
Ratio of the mass of ACV for each product (mean of log-transformed average of the ratio of the geometric mean of duplicates in each subject) after 6 h uptake (filled symbols) and 17 h clearance (open symbols). The shaded area designates the 0.8–1.25 bioequivalence interval used for orally delivered drugs.

The bioequivalence ratios of the mass of ACV in the SC for uptake and clearance are presented in Fig. 5 and Table III. In both Studies 1 and 2, the positive control of the duplicated formulation (US-C+ and AT-C+, respectively) appeared bioequivalent to the corresponding reference formulations (US-Ref and AT-Ref) for uptake and clearance, although the determinations from the traditional bioequivalence analysis were not conclusive (i.e., with the 90% confidence interval lying entirely within the 0.8 to 1.25 window) and would clearly require more than 10 subjects. It also seems, as mentioned above, that the ACV-UK cream is inferior, and ACV-AT is superior, to ACV-US in terms of the drug mass in the SC at both uptake and clearance.

**Table III:** Bioequivalence Assessment between the Products Used in Study 1 (ACV-US versus ACV-UK) and in Study 2 (ACV-AT versus ACV-US). Values are Geometric mean Ratios (Lower - Upper 90% Confidence Interval) for *n* = 10 Subjects

Study 1	US-C+/US-Ref	UK-Test/US-Ref	UK-Test/US-C+	UK-Test/US-Ave ^a^
Uptake	0.87 (0.75–1.01)	0.61 (0.51–0.72)	0.70 (0.58–0.84)	0.65 (0.55–0.77)
Clearance	0.94 (0.76–1.17)	0.58 (0.44–0.76)	0.61 (0.43–0.88)	0.59 (0.44–0.81)
Study 2	AT-C+/AT-Ref	AT-Ref/US-Test	AT-C+/US-Test	AT-Ave/US-Test ^a^
Uptake	1.12 (0.90–1.40)	1.61 (1.22–2.11)	1.81 (1.32–2.48)	1.70 (1.30–2.24)
Clearance	0.86 (0.69–1.07)	1.99 (1.56–2.54)	1.71 (1.41–2.07)	1.84 (1.52–2.23)

^a^Ratios involving US-Ave and AT-Ave were calculated using the geometric mean of 4 replicates (from Ref and C+ combined) for Q_up_ and Q_Cl_ in each subject

The within-subject standard deviation in Q measurements from the ‘reference’ product (*s*_*WR*_) were evaluated for the natural log transformed data as follows (31):

3 $$ {s}_{WR}=\sqrt{\frac{1}{n\left( nr-1\right)}\sum \limits_{j=1}^n\sum \limits_{k=1}^{nr}{\left[\ln \left({\mathrm{Q}}_{jk}\right)-\ln \left({\overline{\mathrm{Q}}}_j\right)\right]}^2} $$

where Q_*jk*_ is the *k*^*th*^ replicate in subject *j*, ${\overline{\mathrm{Q}}}_j$ is the geometric mean of the replicates in subject *j*, *n* is the number of subjects, and *nr* is the number of replicates. For all product comparisons presented in Table III, *s*_*WR*_ > 0.294 (see Table IV), which the FDA identifies as highly variable (31). Sources of this variability could originate in the drug products or the skin-drug product interactions, or be associated with the experimental method (e.g., as typically observed in IVPT studies (16,31)). In a review of data from a previously published SC sampling study of two 1% econazole nitrate generic creams following the protocol used in this study (17,32), we found *s*_*WR*_ of the RLD was less than 0.294, suggesting that the SC sampling method itself is not necessarily highly variable.

**Table IV:** Scaled Average Bioequivalence (SABE) Assessment between the Products Used in Study 1 (ACV-US versus ACV-UK) and in Study 2 (ACV-AT versus ACV-US) for a Bioequivalence Margin (*m*). Products are Considered Bioequivalent if the Upper Bound on the Scaled Average Confidence Interval (SCl_UB_) < 0 and Geometric Mean Ratio (GMR) is within the Limit [1/m, m]

Study 1	Uptake				Clearance			
	*s*_*WR*_	*SCI*_*UB*_		*GMR*	*s*_*WR*_	*SCI*_*UB*_		*GMR*
		*m* = 1.25	*m* = 1.33			*m* = 1.25	*m* = 1.33	
US-C+/US-Ref	0.599	−0.125	−0.235	0.87	0.718	−0.217	−0.372	0.94
UK-Test/US-Ref	0.599	0.200	0.065	0.61	0.718	0.317	0.108	0.58
UK-Test/US-C+	0.321	0.216	0.168	0.70	0.458	0.578	0.466	0.61
Study 2	Uptake				Clearance			
	*s*_*WR*_	*SCI*_*UB*_		*GWR*	*s*_*WR*_	*SCI*_*UB*_		*GMR*
		*m* = 1.25	*m* = 1.33			*m* = 1.25	*m* = 1.33	
AT-C+/AT-Ref	0.695	−0.177	−0.328	1.12	0.426	0.010^*a*^	−0.062	0.86
AT-Ref/US-Test	0.463	0.393	0.295	1.61	0.317	0.787	0.737	1.99
AT-C+/US-Test	0.463	0.658	0.555	1.81	0.317	0.453	0.404	1.71

Given that *s*_*WR*_ >0.294 in this study, we evaluated the mass of ACV in the SC using the reference scaled average bioequivalence (SABE) methodology proposed for assessing highly variable IVPT data (31). In this approach, the test product is considered bioequivalent to the RLD if the geometric mean ratio (GMR) falls within the range [1/*m*, *m*] for the selected bioequivalence margin *m* (currently 1.25 is accepted) and the upper bound of the 90% confidence interval (SCl_UB_) for the quantity, ${\left({\mu}_T-{\mu}_R\right)}^2-{\sigma}_{WR}^2{\left(\ln (m)/0.25\right)}^2$, is less than or equal to zero (where *μ*_*T*_ and *μ*_*R*_ are the population means of the test and reference products, respectively, and ${\mu}_{WR}^2$ is the variance of the reference population, all calculated for the log-transformed data) (16,31). Details of the SABE and the traditional average bioequivalence (ABE) evaluations are provided in the Supplementary Materials along with example calculations for the US-C+ and US-Ref products.

The results of the SABE analysis summarized in Table IV are consistent with the ABE assessment, showing the positive controls to be bioequivalent to the corresponding reference formulations for uptake and clearance for *m* = 1.25, except for ACV-C+ compared with ACV-Ref after clearance, for which SCl_UB_ is almost negative (i.e., it is a very small, although positive number). An analysis of the power curves for these studies (see Supplementary Figs. S8 and S9 and the discussion below) confirms that the study comparing the US-C+ and US-Ref products was adequately powered for a bioequivalence assessment (and successfully demonstrated bioequivalence). By contrast, the study comparing the AT-C+ and AT-Ref products was slightly underpowered for a bioequivalence assessment at *m* = 1.25, but is adequately powered for a bioequivalence assessment at *m* = 1.33 (in which case it successfully demonstrated bioequivalence).

We evaluated the number of subjects required to adequately power the traditional bioequivalence and SABE methods for the *m* = 1.25 and 1.33 limits by performing power simulation studies. For this exercise, the inputs of the power function are the within-subject standard deviation of the reference product, the between-subjects standard deviation, the number of subjects and the number of replicates (see Supplemental Information for more details). This process is repeated 500,000 times under the assumption of bioequivalence. The value of the power is then the percentage of these trials that correctly capture the equivalence relationship between the two products. For *m* = 1.25, the SABE methodology was estimated to achieve a statistical power of at least 80% with 10 subjects for the products compared in Study 1 for both uptake and clearance, and for uptake in Study 2 (which involved a different cohort of 10 subjects) (Supplementary Figs. S5 and S6). Approximately 15 subjects are needed to adequately power the clearance results in Study 2. By comparison, the traditional ABE assessment is estimated to require between 15 and 50 subjects to achieve the same power, with fewer subjects needed in the assessment of the positive control with the corresponding reference product (Figs. S5 and S6). Increasing replication from two to three sites for each product in this study had minimal benefit, reducing the number of subjects required to achieve the same power in the SABE assessment by approximately one subject (Fig. S7).

The fact that ACV-UK and ACV-AT are not bioequivalent with the ACV-US product even at *m* = 1.33 supports a conclusion that the AT-C+ and AT-Ref products did not simply appear to be bioequivalent because the bioequivalence limits were widened to *m* = 1.33 but, rather, because doing so specifically resolved the problem with the power of that study. It should be emphasized that the bioequivalence limits based upon *m* = 1.33 are not currently accepted by the FDA, and it should not be inferred that the Agency is considering any widening of the bioequivalence limits. The analysis performed here, comparing ABE and SABE for both *m* = 1.25 and *m* = 1.33, was only intended to illustrate how the power (and efficiency) of an in vivo stratum corneum sampling bioequivalence study can be greatly increased by an appropriate statistical analysis of the results.

Specifically, the power of a bioequivalence study using cutaneous pharmacokinetic endpoints can be substantially increased by widening the bioequivalence limits from the traditional *m* = 1.25 to *m* = 1.33 for an ABE assessment (Supplementary Figs. S8 and S9) and this means that fewer subjects are needed to power the study, improving efficiency. However, the clear disadvantage of widening the bioequivalence limits is that it essentially lowers the standard for comparability of the test and reference products. In contrast, using an SABE analysis, while maintaining the traditional bioequivalence limit of *m* = 1.25, increases the power of the study to an even greater degree than by widening the bioequivalence limits for an ABE approach to *m* = 1.33. The FDA determined that the marginal additional power gained by an SABE analysis with *m* = 1.33 was not warranted (33) and, as a result, a SABE analysis with *m* = 1.25 was developed and recommended for IVPT studies (9). The analysis performed with the results discussed above agrees with that of the FDA for IVPT studies, and demonstrates that the SABE method can greatly increase the power and efficiency of an in vivo stratum corneum sampling bioequivalence study.

**Fig. 6:**
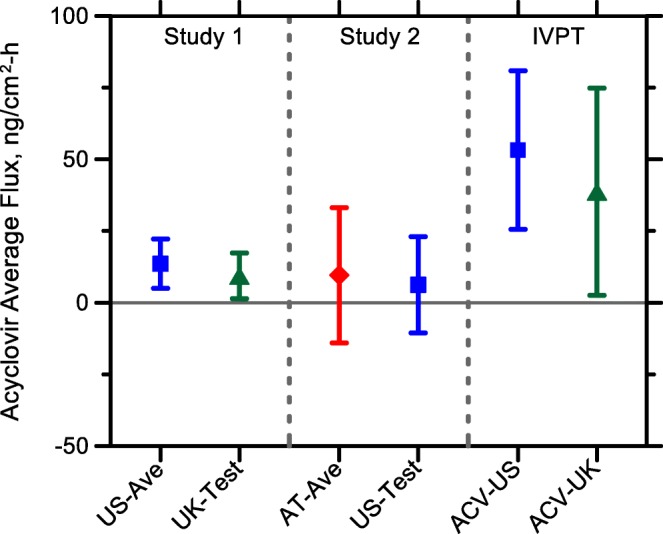
Estimated average in vivo flux of ACV (mean ± 90% confidence interval; n = 10) from the SC into the underlying viable tissue during the clearance period (J_in vivo_) for each cream compared with estimates from IVPT studies (16). US-Ave and AT-Ave were calculated from the difference in the geometric mean of 4 replicates (from Ref and C+ combined) for Q_up_ and Q_Cl_.

Unlike the quantities of ACV measured in the SC, the estimated average fluxes of the drug from the SC into the underlying viable tissue during the clearance period (J_in vivo_) for each cream, in either Study 1 or Study 2 of this work, were not significantly different (Table V and Fig. 6). Similar average fluxes from ACV-US and ACV-UK, calculated over a comparable time interval (8–24 h) after product application, have also been observed in in vitro skin permeation experiments published concurrently with this paper (16) (Fig. 6). Briefly, in this IVPT study, ACV permeation was measured following application of ~15 mg/cm^2^ of cream to dermatomed human skin (4 replicate samples from each of 7 donors) in flow-through diffusion cells. Although, it was not possible to match exactly all features of the in vivo SC sampling and IVPT protocols (e.g., in the IVPT experiments, products were applied by pipette, the ACV-UK product was dispensed from a pump, formulations were not removed during the experiment, and the sample timing was different), there was an evident consistency in the deduced fluxes from the in vivo and in vitro approaches; that is, all are within an order of magnitude which, given the typical variability seen in in vivo and in vitro skin penetration, represents a reasonable level of agreement (Fig. 6). It is worth pointing out that a similar (and even better quantitative) agreement between in vivo SC sampling flux estimates and IVPT measurements has been reported recently for three diclofenac topical products (14).

**Table V:** Comparative Assessment of the Flux And Clearance Rate Constant (k) Calculated as the Difference (mean ± 90% Confidence Interval for *n* = 10 Subjects) between the Products Used in Study 1 (ACV-US versus ACV-UK) and in Study 2 (ACV-AT versus ACV-US)^*a*^

Study 1	US-C+ − US-Ref	UK-Test – US-Ref	UK-Test – US-C+	UK-Test – US-Ave ^*b*^
Flux (ng/cm^2^/h)	−7.8 ± 8.9	−8.1 ± 8.4	−0.31 ± 6.0	−4.0 ± 5.5
10^2^ k (h^−1^)	−0.50 ± 1.4	−0.29 ± 2.3	0.80 ± 2.6	0.54 ± 2.4
Study 2	AT-C+ − AT-Ref	AT-Ref – US-Test	AT-C+ − US-Test	AT-Ave – US-Test ^*b*^
Flux (ng/cm^2^/h)	35.0 ± 38.0	−14.3 ± 37.7	20.7 ± 20.6	−3.3 ± 22.5
10^2^ k (h^−1^)	1.6 ± 1.8	−1.3 ± 2.2	0.33 ± 1.6	0.46 ± 1.7

^a^Differences between the designated products were not significantly different from zero in any case (*p* < 0.05)

^b^Flux and k values for US-Ave and AT-Ave were calculated using the geometric mean of the 4 replicates (from Ref and C+ combined) for Q_Up_ and Q_Cl_

While a similar comparison between the Study 2 in vivo results and IVPT data is not possible because of insufficient in vitro information being available for ACV-AT, the latter formulation and ACV-US have been investigated in an elegant series of in vivo dermal open-flow microperfusion (dOFM) experiments (12). Two dOFM probes were inserted in each of 6 treatment sites, 3 sites/thigh, to monitor the intradermal ACV concentration as a function of time from the duplicate application of ACV-AT and 2 x ACV-US in 20 healthy subjects. The average drug concentrations measured in the dOFM perfusate, in the 4- to 24-h period post-treatment, were 0.70 and 0.64 ng/mL for ACV-AT and ACV-US, respectively, with an average standard error of the mean (for both products) of 0.13 ng/mL.^1^ As these concentrations must be proportional to the flux at which the drug is arriving in the viable epidermis, it follows that these dOFM results are consistent with the SC sampling estimates of flux (Table II and Fig. 6) and demonstrate that the rates of ACV delivery from these two creams are not significantly different. However, this observation is at odds with the conclusion of the dOFM paper, which used the 0- to 36-h area under the perfusate concentration of ACV versus time profile, and the maximum ACV concentration in the perfusate, as the metrics for assessing bioequivalence between the products studied. This divergence points to important issues requiring further examination as potential surrogate measures of topical bioequivalence emerge and the development of in vitro*-*in vivo correlations are sought. For instance, should the selection of an alternative approach be solely determined on the rigour with which the method can be applied (e.g., an IVPT experiment run for 48 h under closely controlled conditions) or should precedence be given to a technique that is best capable of assessing bioequivalence under conditions that are closest to real-world use (such as SC sampling in the case of antiviral treatment of cold sores, where a dose interval may be only a few hours)?

The flux of the drug from the SC into the underlying viable tissue depends on both the amount of drug in the SC (i.e., Q_Up_) as well as the diffusion rate through the SC, which is evident from the relationship between J_in vivo_ and the first-order clearance rate constant (k) over the time interval Δt, i.e.:

4 $$ {\mathrm{J}}_{\mathrm{in}\ \mathrm{vivo}}={\mathrm{Q}}_{\mathrm{Up}}\ \left[1\hbox{--} \exp \left(-\mathrm{k}\ \Delta \mathrm{t}\right)\right]/\Delta \mathrm{t} $$

Therefore, comparison of products in terms of both J_in vivo_ and k offers the possibility of identifying the underlying mechanism of any difference observed. For example, when a difference between J_in vivo_ values is observed without a corresponding difference in k, it is likely that the drug’s partitioning into the SC has changed rather than its diffusion through the barrier. The physical distinction between J_in vivo_ and k is important to emphasise. The flux is a tangible and model-independent parameter reporting on the quantity of drug that is entering the viable skin layers below the SC over a certain time period. Like the measurement of Q_Up_, for example, J_in vivo_ offers a metric that can be used to distinguish between different formulations and to assess their equivalence. The quantity of drug taken up into the SC, and the flux into the underlying tissue, would be expected to link to the thermodynamic activity of the active in the formulation. In contrast, the apparent 1st-order rate constant k is a fitted parameter to a pre-defined model (for which full validation is not perhaps currently at hand). A clear advantage of k is its dose-independence (assuming the boundary conditions of the model are satisfied) and that its absolute value says something quite specific about the rate at which drug is ‘cleared’ from the SC; for example, if k = 0.3 h^−1^, then 30% of the drug in the SC at any moment will have left in the following period of 1 h. In this work, the deduced values of J_in vivo_ and k, in both Study 1 and Study 2, were not significantly different between the different creams considered (Tables II and V), despite the fact that the ACV-UK cream was inferior, and ACV-AT superior, to ACV-US in terms of the drug mass in the SC at both uptake and clearance. A plausible explanation for this observation is that the 10 subjects involved in each study (and the resulting high level of variability in the calculated J_in vivo_ and k values) were insufficient to provide the necessary statistical power.

The calculated in vivo flux of ACV into the viable epidermis represents a key piece of information necessary to determine whether, if sustained, the delivery would permit a local, effective concentration of the drug to be achieved. Indeed, this idea has previously been explored for ACV in some depth by Higuchi and colleagues (34–38) who proposed the so-called “C* Concept” as an approach to relate the free drug concentration at the site of action (C*) to in vivo efficacy. The model essentially links the drug flux (J in units of amount per unit area per unit time) to C* (amount per volume) at the target site, assumed for ACV to be the basal epidermis, and a heterogeneous rate constant (P_D_ with dimensions of distance per time) describing drug clearance from the site; that is

5 $$ \mathrm{J}={\mathrm{P}}_{\mathrm{D}}\ \mathrm{x}\ \mathrm{C}\ast $$

The “C* Concept” is a steady-state representation, therefore, and P_D_ indicates that the loss of drug away from the site of action occurs via passive diffusion into the dermis, where uptake by the microcirculation provides a perfect sink. It follows that:

6 $$ {\mathrm{P}}_{\mathrm{D}}={\mathrm{D}}_{\mathrm{D}}/{\mathrm{h}}_{\mathrm{D}} $$

where D_D_ is the drug diffusivity in the dermis and h_D_ is the diffusion path-length from the basal epidermis to the microcirculation where the drug is irreversibly removed from the skin.

Although the in vivo fluxes deduced from the experiments reported here were not determined at steady-state (and are likely to be underestimates, therefore – see below), they do provide ‘ball-park’ starting values for the purpose of illustration. To estimate C* requires a value for P_D_ (or for the composite parameters, D_D_ and h_D_) and 1.4 × 10^−3^ cm s^−1^ (~5 cm h^−1^) was originally proposed for ACV (35). This was based on an in vitro measurement of the permeability coefficient of ACV across heat-separated dermis multiplied by 20 to take into account the shorter diffusion path-length in vivo (i.e., the distance the drug would travel before interception by microcirculation uptake).

An alternative approach to the estimation of P_D_ involves separate derivation of appropriate values of D_D_ and h_D_. In terms of the diffusion path-length (h_D_) from the basal epidermis to the superficial papillary plexus of blood vessels in the dermis, histological evidence suggests that this is likely to be no more than 100 μm (which would correspond to about 1/10th of the average dermal thickness) (39). The multiplier of 20 used earlier (35) implies a similar value of 50 μm. With respect to the drug’s dermal diffusivity, there are both experimental and theoretical approaches available for its assessment. Broadly speaking, the dermis has been likened in terms of consistency to a hydrogel-like matrix such that D_D_ values would be expected to be up to an order of magnitude lower than those in water (40,41). More rigorously, based on available literature data (measured at or adjusted to 37°C) combined with mathematical modelling, Kretsos et al. (40) have derived simple empirical equations with which to estimate D_D_ (in cm^2^ s^−1^), including:

7 $$ \log\ {\mathrm{D}}_{\mathrm{D}}=-4.15\hbox{--} \left(0.655\times \log\ \mathrm{MW}\right) $$

8 $$ \log\ {\mathrm{D}}_{\mathrm{D}}=-4.38\hbox{--} \left(0.207\times {\mathrm{MW}}^{1/3}\right) $$

where MW is the drug’s molecular weight in Daltons. For ACV, Eqs. (7) and (8) predict dermal diffusion coefficients of the free drug of 2.04 × 10^−6^ cm^2^ s^−1^ and 2.31 × 10^−6^ cm^2^ s^−1^, respectively, values approximately 3- to 4-fold less than those expected in water.

Taking the average of these estimates for D_D_ and setting h_D_ to 100 μm yields a value of P_D_ of about 0.78 cm h^−1^. From Table II, taking the results from all the ACV products tested, the average value of J was 10.3 ng cm^−2^ h^−1^, permitting C* to be calculated from the re-arranged form of Eq. (5) (i.e., C* = J/P_D_) as ~13 ng/mL.

As mentioned before, this predicted C* will inevitably be lower than the actual steady-state level in the viable epidermis due to the relatively short duration of the experiment. However, from the SC sampling data, it is possible (as has been demonstrated in the literature (17) and is summarised in the Appendix) to deduce the lag-time for ACV diffusion and from this both to determine when steady-state would, under normal circumstances, be reached and then to extrapolate from the estimated C* above to the ‘real’ value. When this is done, the predicted C* at a nominal steady-state is close to 40 ng/mL (i.e., the steady-state flux across the skin is predicted to be about 3-fold higher than that measured by SC sampling). Regardless of the manner in which C* is evaluated here, however, the result is much less than the reported effective concentration which, in hairless mouse, was 100 to 1000 ng/mL (0 to 100% efficacy) (37). As this represents the local tissue concentration of the drug, a similar value might reasonably be expected to apply in humans as well.

The extent to which this observation may call into question the therapeutic efficacy of the ACV products tested is not a subject that can be addressed here as no pharmacological measurements were made in this research. However, the credence of the estimation of C* using the SC sampling method can be evaluated by comparison with the published dOFM results. As mentioned above, these experiments were carried out over a 36-h period, by the end of which the ACV concentration in the perfusate had been relatively constant for 12 h at a value of roughly 1 ng/mL. As the flux of drug into the perfusate should be proportional to its concentration in the tissue, and as it would appear that steady-state had been reached, this flux should also reflect the rate at which ACV is entering the tissue itself (i.e., equivalent to J in Eq. (5) above). The flux of ACV into the perfusate in the dOFM experiment is the flow rate through the dialysis fibre (60 μL/h) multiplied by the drug concentration in the perfusate (i.e., ~1 ng/mL), and this works out to be approximately 0.06 ng/h. To express this value normalised per unit area must consider the geometry of the dOFM probe, the planar projection of which is a rectangle of 15 mm (the length of the probe) by 0.5 mm (the probe’s diameter) corresponding to an area of 0.075 cm^2^. Thus, it follows that J can be estimated to be about 0.8 ng/cm^2^/h assuming perfect unimpeded collection into the fibre from the surrounding tissues; that is, roughly an order of magnitude of the average of the values determined by SC sampling in Table II, and a factor ~40-fold smaller than the extrapolated steady-state flux (see Appendix and Table VI for details).

**Table VI:** Steady-State Flux (J_*ss*_) Predicted from Calculations Prescribed by Eqs. (A1)–(A4) ^*a*^

Quantity	Units	Value
Q_Up_	μg cm^−2^	0.87 (0.74–1.02)
Q_Cl_	μg cm^−2^	0.69 (0.58–0.83)
*W*	unitless	0.79 (0.72–0.88)
*t*_*lag*_	h	18.9
*t*_*Up*_/*t*_*lag*_	unitless	0.3
*F*	unitless	1.93
Q_ss_	μg cm^−2^	1.68
J_*ss*_	ng cm^−2^ h^−1^	29.6

^a^Values for Q_Up_, Q_Cl_ and *W* are the log mean average (90% CI) of the geometric mean of duplicates for the Ref, C+, and Test products in both studies combined, *n* = 60. All other values were calculated using the average values for Q_Up_, Q_Cl_ and *W*

In broad terms, therefore, the results, observed and derived, from the SC sampling and dOFM experiments are consistent with one another, and both indicate C* levels of ACV that are at least 25-fold smaller than those reported to be therapeutically effective. Given the information presently available, it is not possible to definitively explain why the SC sampling approach resulted in more ACV reaching the viable epidermis than that deduced from the dOFM experiments, although a collection efficiency by the dOFM fibres of less than 100% is likely an important factor. It is also possible that the more vigorous massaging used in the SC sampling study^2^ may have altered the dynamics of the formulation metamorphosis (42) during the initial period post-application, such that more ACV is transferred into the SC more quickly before the loss of co-solvents causes the subsequent delivery across the residual phase-SC interface to significantly decelerate. Then, even though the formulations remain in contact with the skin in the dOFM studies (whereas they are removed at 6-h in the SC sampling method), the flux over the majority of the 36-h experimental duration is substantially attenuated.

## Conclusions

While the two component studies described in this paper were not sufficiently powered for a meaningful, regulatory-standard bioequivalence assessment between the drug products examined, the two-point SC sampling procedure did reveal some statistically significant differences. The robustness of the method was demonstrated by the consistency in the results for ACV-US (which was used in both Studies 1 and 2) that were acquired by multiple investigators. Incorporation of a ‘positive control’ by duplicating measurements of the “reference” product in each study contributed further validation of the approach. The within-subject variability of drug amounts in the SC from the reference products exceeded the standard cut-off, indicating use of the scaled average bioequivalence (SABE) methodology. The SABE assessment offered significant advantages over the traditional bioequivalence methodology in achieving adequate power with fewer subjects.

The comparison of the SC sampling results with the previously published (12) dOFM measurements for the ACV-US and ACV-AT creams raises issues worthy of additional consideration. There is good overlap between the two sets of data when the assessment is made over the same period of time post-application of the drug products; this is not the case when the dOFM findings are analysed over the entire duration of those experiments (12). With respect to any future bioequivalence assessment protocol design, therefore, and regardless of the surrogate method used (including IVPT), it is appropriate to ask whether the approach should simply maximise the quality of the information obtainable, or whether the comparison should be made under conditions as close as possible to those in which the product is to be used?

As has been previously reported (13,14), SC sampling can be used to calculate the delivery rate of a drug to the skin ‘compartment’ below the barrier (i.e., the viable epidermis – upper dermis) either empirically as a flux, in amount per unit time per unit area, or as a fitted, dose-independent, 1st-order rate constant. These metrics, in the work described here, were subject to variability and, given the limited number of repeat measurements made, were not significantly different across the three formulations considered.

Finally, from the derived flux, it was possible to use the C* Concept model (34–38), originally proposed more than 20 years ago, to predict the ACV concentration in the viable skin ‘compartment’ from the quantities of drug measured in the SC. This estimate of the ACV level at, or at least near to, its site of action was well below that believed to be therapeutically effective (37) but agreed, to within an order of magnitude, with recently published dOFM data in humans (12).

Taken together, this research with acyclovir as the prototypical drug demonstrates that SC sampling is a simple, minimally invasive, in vivo approach to the assessment of topical bioequivalence that not only (a) complements and reinforces the teachings of other methodologies, but also (b) provides a path by which information pertinent to the bioavailability of an active pharmaceutical ingredient in the skin below the SC can be revealed and local concentrations at sites of action in the viable tissue may be estimated.

### Electronic supplementary material

ESM 1 (PDF 1077 kb)

## Appendix

The steady-state flux of drug J_ss_ through a homogeneous membrane with a constant concentration at the surface layer of the membrane is described by:

A1 $$ {\mathrm{J}}_{\mathrm{ss}}={\mathrm{Q}}_{\mathrm{ss}}/\left(3\ {t}_{lag}\right) $$

where the steady-state mass of drug per unit area in the membrane Q_ss_ = *C*_*o*_ *L*/2. The lag time *t*_*lag*_ for diffusion through the membrane can be estimated from the ratio (*W*) of the mass in the membrane at clearance relative to uptake. If the uptake time *t*_*Up*_ is too short to achieve steady-state, then *t*_*lag*_ can be estimated by solving the following expression (17):

A2 $$ \hbox{--} {\mathit{\log}}_{10}W\hbox{--} 1.072\ \left(t\hbox{--} {t}_{Up}\right)/\left(6\ {t}_{lag}\right)+{\mathit{\log}}_{10}\left(0.978\hbox{--} 0.133\ {\mathit{\log}}_{10}\left[{\mathrm{t}}_{\mathrm{Up}}/\left(6\ {t}_{lag}\right)\right]\right)=0 $$

and then used to estimate Q_ss_ from the mass of drug per unit area in the membrane at uptake (Q_Up_):

A3 $$ {\mathrm{Q}}_{\mathrm{ss}}=F\ {\mathrm{Q}}_{\mathrm{Up}} $$

where *F* is the ratio of the average concentration in the membrane at steady-state to that at *t*_*Up*_ which is calculated from (43):

A4 $$ 1/F=1\hbox{--} \left(8/{\uppi}^2\right)\ \sum \limits_{n=0}^{\infty}\mathit{\exp}\left[-{\left(2n+1\right)}^2\ {\uppi}^2\ \left({t}_{up}/6\ {t}_{lag}\ \right)\right]/{\left(2n+1\right)}^2 $$

Equation (A2) provides reasonable estimates of *t*_*lag*_ for 0.06*t*_*lag*_ < *t*_*Up*_ < 1.2*t*_*lag*_. Table VI summarizes the average SC sampling data and the estimated *t*_*lag*_ for ACV clearance and J_ss_.

The assumption of constant concentration at the surface layer of the membrane *C*_*o*_ requires the thermodynamic activity of the drug at the SC surface to be constant during the exposure; i.e., metamorphosis of the formulation as components evaporate and absorb should have a minimal effect. In actual practice, the recommended, frequent, repeat dosing of the formulation may approximately achieve this scenario even though it probably does not occur for a single application. Certainly, in other cases (e.g., econazole (32)), there is evidence that the transformation of a formulation post-application to the skin, and the differential rates of disposition of the drug and excipients – from a single treatment – eventually causes the continued uptake of the active over time to cease; that is, once volatile components of the formulation and the more mobile co-solvents have been lost either by volatilization and/or skin penetration, the drug may no longer remain solubilized and be unable, therefore, to diffuse into and through the SC. It follows that the estimation of J_ss_ in all likelihood represents an upper limit on the true value that would be achieved in a realistic dosing scenario; the actual flux will probably fall, therefore, between that deduced from the SC sampling experiment (Table II) and the predicted value from the calculations outlined above.

## Abbreviations

ABE: Average bioequivalence
ACV: Acyclovir
ACV-AT: Aciclovir 1A Pharma Cream (1A Pharma GmbH, Vienna, Austria)
ACV-UK: Zovirax® (GlaxoSmithKline Consumer Healthcare, Brentford, UK)
ACV-US: Zovirax® (Valeant Pharmaceuticals, Bridgewater, US)
C*: The free drug concentration at the site of action
*C*_*o*_: Concentration at the surface layer of the membrane
D_D_: Drug diffusivity in the dermis
dOFM: dermal open-flow microperfusion
*F*: Ratio of the average concentration in the membrane at steady-state to that at *t*_*Up*_
FDA: United States Food & Drug Administration
GMR: Geometric mean ratio
h_D_: Diffusion path-length from the basal epidermis to the microcirculation
IVPT: In vitro permeation test (using excised skin mounted on a diffusion cell)
J_in vivo_: Flux of drug out of stratum corneum into underlying tissue
J_ss_: Steady-state flux of drug through a homogeneous membrane
k: 1st-order rate constant describing clearance from the stratum corneum
*L*: Diffusion path-length through (or thickness of) a membrane
*m*: Bioequivalence margin
MW: Molecular weight (Daltons)
P_D_: Heterogeneous rate constant describing drug clearance from the site of action
Q_Up_: Mass per unit area of drug in the stratum corneum at t_Up_
Q_Cl_: Mass per unit area of drug in the stratum corneum at t_Cl_
Q_ss_: Steady-state mass of drug per unit area in the membrane
RLD: Reference Listed Drug
SABE: Scaled average bioequivalence
SC: Stratum corneum
*s*_*WR*_: Within-subject standard deviation in Q measurements from the ‘reference’ product
t_lag_: Time lag for diffusion through a membrane
t_Up_: Uptake time
t_Cl_: Clearance time
Δt: Time between t_up_ and t_Cl_
US: United States of America
W: Ratio of the mass in the membrane at clearance relative to uptake

## Footnotes

1 Determined by digitizing Figure 3 from Bodenlenz et al. (12) using Grapher Version 11 (Golden Software, Golden, Colorado, US).

2 F. Sinner, in a personal communication, reported that the application of the formulations in the dOFM experiments involved the gentlest of massaging so as to avoid perturbation of the microdialysis fibres situated, of course, just below the skin surface.
