# Supplementary material for: Stratum Corneum Sampling to Assess Bioequivalence between Topical Acyclovir Products
Source: Pharm Res. 2019 Nov 14;36(12):180. doi: 10.1007/s11095-019-2707-3 (PMC6856025; doi:10.1007/s11095-019-2707-3)
Supplement: Supplementary file 1 — (PDF 1077 kb) [file 11095_2019_2707_MOESM1_ESM.pdf]

Supplementary Information for the paper:

Stratum corneum sampling to assess bioequivalence between topical acyclovir products

A. Pensado[1], W.S. Chiu[1], S.F. Cordery[1], E. Rantou,[2]
A.L. Bunge[3], M.B. Delgado-Charro[1] and R.H. Guy[1,4]

[1] Department of Pharmacy & Pharmacology, University of Bath, Bath, UK

[2] Office of Biostatistics, Office of Translational Sciences, Center for Drug Evaluation and Research, United States Food and Drug Administration, Silver Springs, MD, USA

[3] Chemical and Biological Engineering, Colorado School of Mines, Golden, Colorado, USA

[4] To whom correspondence should be addressed at: Department of Pharmacy & Pharmacology, University of Bath, Claverton Down, Bath, BA2 7AY, U.K.; email: r.h.guy@bath.ac.uk

Additional results

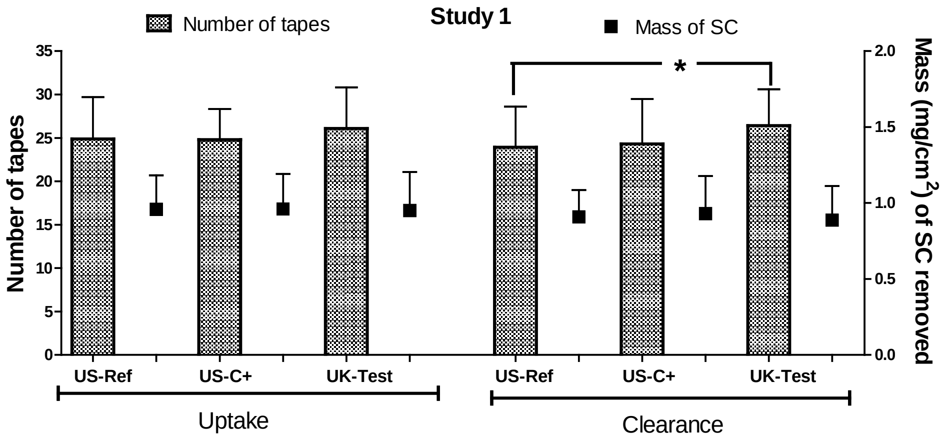

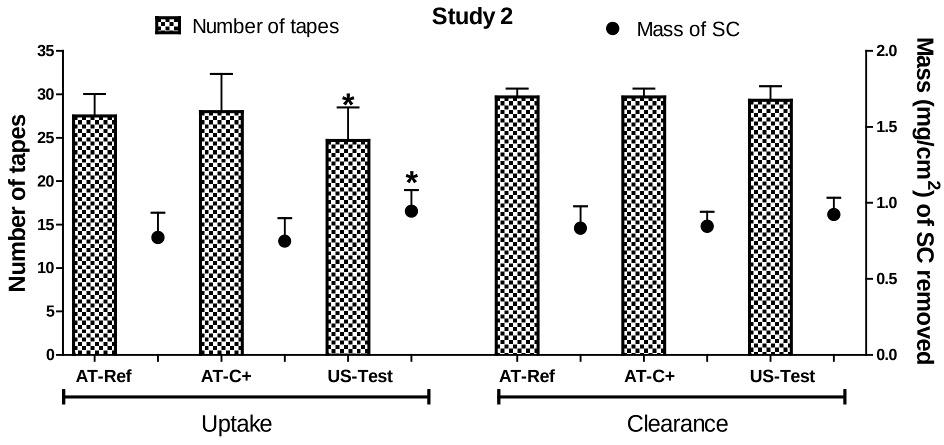

Figure S1. Number of tapes (columns) collected and mass of SC removed (symbols) from each subject for three ACV creams after 6 h uptake and 17 h of clearance. The results are represented as the arithmetic mean and standard deviation of the arithmetic average of the duplicate measurements in 10 subjects for each of the three products after uptake and clearance. *Significantly different from other cream applications measured after uptake or clearance in the same study ($p<0.05$). Notes: [1] The geometric mean for the duplicate measurements of the mass of drug in the SC is used because we expect (based on other skin permeation measurements) that this is likely to be log-normally distributed. However, it is not clear that the number of tape-strips, or the mass of SC collected on the tape-strips, would be expected to be log-normally distributed. It therefore makes more sense to use the arithmetic average of the duplicates. In practice, though, this changes minimally the numerical value of the specific data points which are plotted above. [2] For Study 2, clearance, the maximum permitted number of tape-strips (30) were taken at 57 of 60 sites. In contrast, in Study 2, uptake, and in Study 1, uptake and clearance, at only about half of the sites were 30 tape-strips removed (27 for uptake and clearance in Study 1, and 36 for uptake in Study 2).

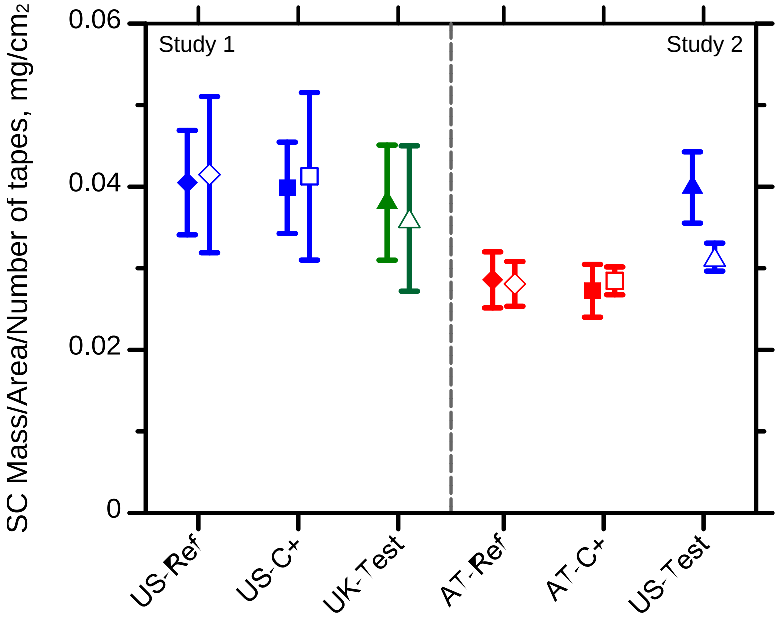

Figure S2. Average SC collection efficiency of the tape strips (mg/cm2) for each cream (arithmetic mean ± 90% confidence interval for 10 subjects) after 6 h uptake (filled symbols) and 17 h clearance (open symbols).

These results and the methodology employed indicate some possible procedural recommendations that might be introduced to ensure the uniformity and reproducibility of the approach. First, the refined SC sampling technique introduced in the econazole work (1) did not measure the mass of SC on the tapes. However, a pilot study was performed that employed TEWL measurements to decide if tape-stripping had completely and reliably removed most of the SC; hence, it was unnecessary to measure the mass of SC on the tapes. A sensible suggestion, therefore, would be to quantify the amount of SC removed unless a pilot study (using TEWL, for example) had verified that the method did indeed ensure that most of the barrier had been collected.

Second, the improved protocol (1) specified that 12 tape-strips should always be taken (in fact, no TEWL values were recorded in that study until 12 tape-strips had been removed). In the present work, at only one site, on a single volunteer, were less than 12 tape-strips acquired before the procedure was stopped. A reasonable recommendation would therefore be to set a 12 tape-strip minimum for SC sampling. Nonetheless, attention should always be paid to the nature and quantity of the excipients present in the drug products tested, as there will certainly be some cases (see, for example, (2) where a formulation alters the SC and dramatically increases the amount of tissue collected). In such circumstances, to spare volunteers from inordinate discomfort, acquisition of less

than 12 tape-strips would be appropriate. A pilot study, once again, may be used to guide protocol development accordingly.

Third, the sufficiency of the 30 tape-strip maximum for the tape we have been using, may be questioned given that the TEWL criterion for stopping SC removal had not been met at about 75% of the treated skin sites. This points out the value of measuring the SC mass removed as the quantities acquired demonstrated that most of the SC had indeed been collected; such information would be revealed, of course, in a sensibly configured pilot study.

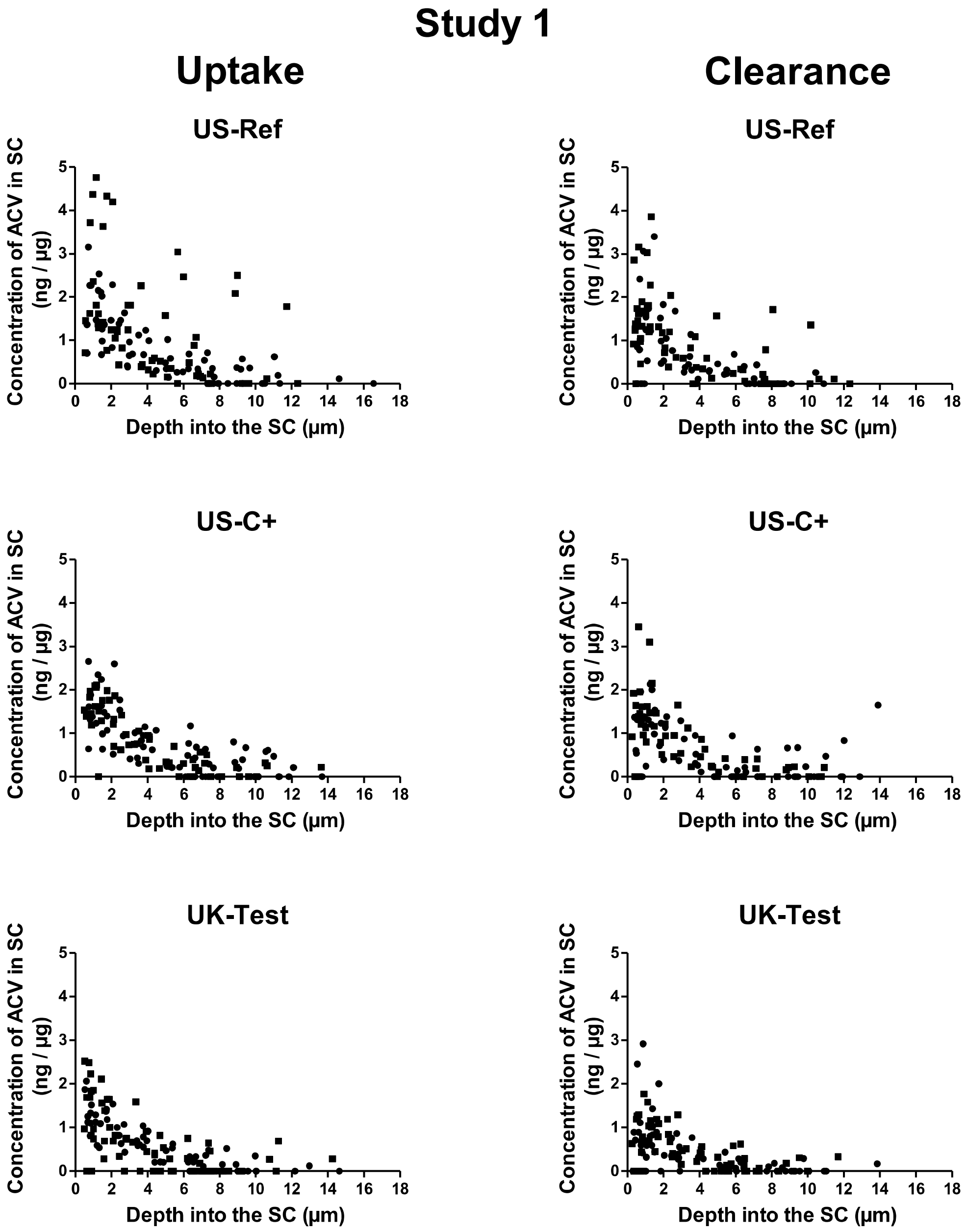

Figure S3. Concentration of ACV (ng/µg of SC) recovered in Study 1 from the tapes in 10 subjects after 6 h uptake and 17 h clearance for US-Ref, US-C+ and UK-Test creams. Figures show profiles of drug concentration versus depth in the SC; raw data (not averages of duplicate values) are plotted.

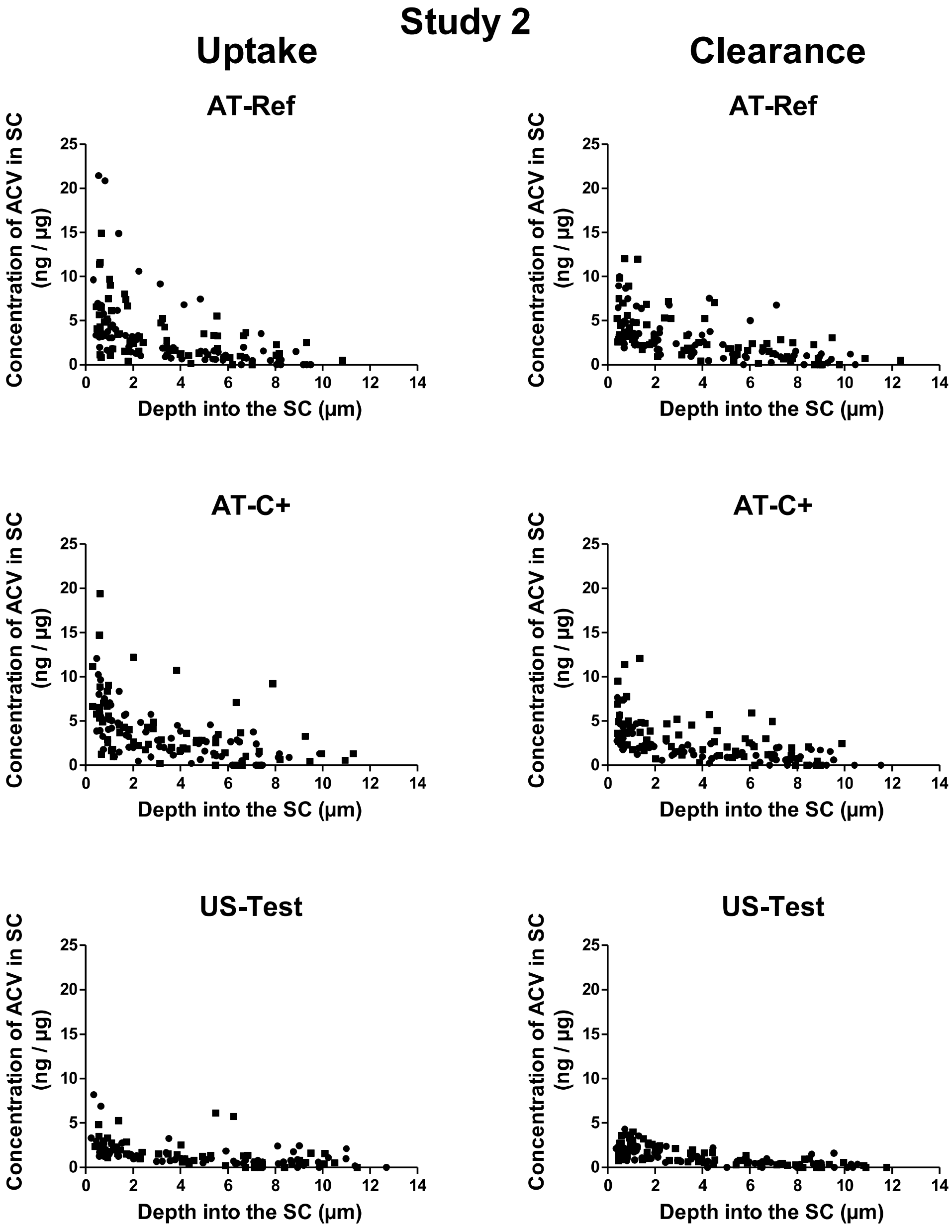

Figure S4. Concentration of ACV (ng/µg of SC) recovered in Study 2 from the tapes in 10 subjects after 6 h uptake and 17 h clearance for AT-Ref, AT-C+ and US-Test creams. Figures show profiles of drug concentration versus depth in the SC; raw data (not averages of duplicate values) are plotted. Note the y-axis maximum is 5-fold larger than in Figure S3. The US-Test results in Study 2 are consistent with the US-Ref and US-C+ results presented in Figure S3. The concentration profiles for the ACV-AT products were clearly different than that observed for the ACV-US cream after both uptake and clearance.

Data analysis and example calculations

Calculated metrics

The mass per unit area of drug in the tape strips was determined in the kth replicated site treated with formulation *i* on subject *j* (Q_{ijk}). Each formulation in the two studies was measured at 2 sites in 10 subjects; i.e., *nr* = 2 and *n* = 10. The first-order clearance rate constant (k) and the flux of drug out of the SC into the underlying tissue $\text{J}_{\text{in vivo}}$ were calculated from the geometric mean of the duplicate ($\bar{Q}_{ij.}$) calculated as follows:

$Z_{ijk} = \ln\left(Q_{ijk}\right)$ = natural log transformed value of Q_{ijk} for formulation *i* tested in the kth replicated site on subject *j*

$\ln\left(\bar{Q}_{ij.}\right) = \bar{Z}_{ij.} = \frac{1}{nr}\sum_{k=1}^{nr} Z_{ijk}$ = average of the log-transformed Q_{ijk} for formulation *i* in each subject *j*

$\bar{Q}_{ij.} = \exp\left(\bar{Z}_{ij.}\right)$ = geometric mean of the metric for formulation *i* in each subject *j*

Values for $\text{J}_{\text{in vivo}}$ and k were calculated for formulation *i* in subject *j* using $\bar{Q}_{ij.}$ determined at the end of the uptake period ($\bar{Q}_{up,ij.}$) and after a period of clearance following the uptake period ($\bar{Q}_{cl,ij.}$) as follows

$$\text{J}_{\text{in vivo},ij} = \left(\bar{Q}_{up,ij.} - \bar{Q}_{cl,ij.}\right)/\Delta t$$

$$\text{k}_{ij} = \ln\left(\bar{Q}_{up,ij.} - \bar{Q}_{cl,ij.}\right)/\Delta t$$

Because $\text{J}_{\text{in vivo}}$ and k can exhibit both positive and negative values, log-transformation is not appropriate. Example calculations for the US-Ref and US-C+ formulations are listed for $\bar{Q}_{ij.}$ after uptake and clearance (Table S1) and for $\text{J}_{\text{in vivo},ij}$ and k_{ij} (Table S2).

Bioequivalence assessment

Bioequivalence calculations for a balanced study design with *nr* replicated measurements of each formulation in a total of *n* subjects were conducted using the following procedures for a metric determined in the kth replicated site treated with formulation *i* on subject *j* (M_{ijk}).

For M_{ijk} representing a metric that is always a positive number (i.e., the mass per unit area of drug collected in the SC tape strips, Q_{ijk}) the BE analysis is performed by comparing the log-transformed value of M_{ijk}, defined as Z_{ijk}, determined in each subject and then averaging across subjects as follows:

$Z_{ijk} = \ln\left(M_{ijk}\right)$ = natural log transformed value of M_{ijk}

$\bar{Z}_{ij.} = \frac{1}{nr}\sum_{k=1}^{nr} Z_{ijk}$ = mean of the log-transformed metric for formulation *i* in each subject *j*

$\bar{M}_{ij.} = \exp\left(\bar{Z}_{ij.}\right)$ = geometric mean of the metric for formulation *i* in each subject *j*

$\bar{I}_j = \bar{Z}_{1j.} - \bar{Z}_{2j.}$ = difference between formulations 1 and 2 of the mean of the log-transformed metric in each subject *j*

$\bar{I} = \frac{1}{n}\sum_{j=1}^{n} \bar{I}_j$ = mean of the differences between formulations 1 and 2 of the log-transformed metric averaged over *n* subjects

$s_I^2 = \frac{1}{n-1}\sum_{j=1}^{n}\left(\bar{I}_j - \bar{I}\right)^2$ = inter-subject variance of the differences between formulations 1 and 2 of the log-transformed metric

The within subject variance for a formulation *i* measured in subject *j* is calculated as follows:

$s_{Wi}^2 = \frac{1}{n\left(nr-1\right)}\sum_{j=1}^{n}\sum_{k=1}^{nr}\left[Z_{ijk} - \bar{Z}_{ij.}\right]^2$ = within subject variance of the log-transformed metric for formulation *i*

Note that for a balanced study design, estimates for $\bar{I}$ and s_I are the same for $\bar{I}_j$ calculated from the difference of the mean of the log-transformed metric (i.e., $\bar{I}_j = \bar{Z}_{1j.} - \bar{Z}_{2j.}$) or calculated as the mean of randomly matched replicates (i.e., $\bar{I}_j = \frac{1}{nr}\sum_{k=1}^{nr}\left(Z_{1jk} - Z_{2jk}\right)$ as specified in Rantou et al. (3). Example calculations of the BE calculation methodology comparing the US-C+ formulation to the US-Ref formulation are presented in Table S3. Example calculations of the within subject variance for the US-Ref formulation are listed in Table S4.

Traditional average bioequivalence (ABE) assessment

In the traditional ABE methodology, formulations 1 and 2 are considered bioequivalent for a margin *m* if:

$$\left|\overline{I} \pm \delta_{I,90\%}\right| \leq \ln(m)$$

$\delta_{I,90\%} = \dfrac{s_I \cdot t_{0.95,n-1}}{\sqrt{n}}$ = projected half-width of the 90% confidence interval (CI) for the population mean difference (4)

where $t_{0.95,n-1}$ is the 95th percentile of the Student's T-distribution with n-1 degrees of freedom. Consistent with tradition, in the calculations presented here m = 1.25, which corresponds to the BE interval [0.8, 1.25].

Results are presented as the geometric mean ratio (*GMR*) of the selected metric for formulation 1 compared with formulation 2 and the projected lower and upper 90% CIs for the population mean ratio ($GMR_{90\%,upper}$ and $GMR_{90\%,lower}$ $R_{90\%,lower}$, respectively) calculated as:

$$GMR = \exp\left(\overline{I}\right) \qquad GMR_{90\%,lower} = \exp\left(\overline{I} + \delta_{90\%}\right) \qquad GMR_{90\%,upper} = \exp\left(\overline{I} - \delta_{I,90\%}\right)$$

Example calculations of the traditional BE assessment for the US-C+ formulation compared to the US-Ref formulation are provided in Table S3.

Note that calculated values for the *GMR* and the lower and upper confidence intervals are the same for data transformed using natural logarithms (as described here) or base 10 logarithms (as described previously (1)) provided the anti-log step is consistent with the type of log transformation (i.e., exp(x) for natural log transformed data and 10^x for base 10 log transformed data). Here, we recommend natural log transformation for the ABE calculations to be consistent with the scaled average bioequivalence (SABE) procedure below, which is specific to the type of logarithmic transformation.

<u>Scaled average bioequivalence (SABE) assessment</u>

The SABE methodology is indicated when the within subject standard deviation for the reference formulation (i.e., s_{Wi} for i = 2 in an assessment of the difference between formulations 1 and 2 for the natural log-transformed metric) is

$$s_{W2} > 0.294$$

In this case, the formulation 1 is considered bioequivalent to the formulation 2 if the geometric mean ratio (*GMR*), calculated as described above, falls within the range [1/*m*, *m*] for the selected bioequivalence margin *m* (currently 1.25 is accepted) and the upper 95% confidence interval (SCI_{UB}) for the quantity, $\left(\mu_1 - \mu_2\right)^2 - \sigma_{W2}^2\left(\ln(m)/0.25\right)^2$, is less than or equal to zero (where μ_1 and μ_2 are the population means

of the test and reference products, respectively, and σ_{W2}^2 is the variance of the reference population, all calculated for the log-transformed metric) (3,5). Calculation of SCI_{UB} is described by Rantou *et al.* (3) and summarized below

$$\theta = \left[\ln(m)/\sigma_{W0}\right]^2$$

$$X = \overline{I}^2 - s_I^2/n$$

$$Y = -\theta s_{W2}^2 ,$$

$$X'_\beta = \left(\left|\overline{I}\right| + t_{0.95,\, n-1}\sqrt{s_I^2/n}\right)^2$$

$$Y'_\beta = -\theta \frac{n(nr-1)s_{W2}^2}{\chi^2_{0.95,n(nr-1)}}$$

$$V = \left(X'_\beta - X\right)\left|X'_\beta - X\right| + \left(Y'_\beta - Y\right)\left|Y'_\beta - Y\right|$$

$$SCI_{UB} = X + Y + \left(|V|/V\right)\sqrt{|V|}$$

where σ_{W0} a regulatory constant (set equal to 0.25 in these calculations), $t_{0.95,n-1}$ is as defined for the traditional ABE assessment and $\chi^2_{0.95,nr(n-1)}$ is the 95th percentile of the Chi-Square distribution with *n*(*nr*-1) degrees of freedom. Example SABE calculations for the US-C+ formulation compared to the US-Ref formulation are listed in Table S5. Note that the SABE calculations presented here (and described in Rantou *et al.* (3)) assume the assessed metric was transformed using the natural log. If base-10 log transformations are used, then the SABE methodology is indicated when the within subject standard deviation for the base-10 log transformed metric ($s_{W2,\log 10}$) is

$$s_{W2,\log 10} > 0.294/\ln(10) = 0.294/2.303 = 0.128$$

and

$$SCI_{UB} = \left[\ln(10)\right]^2 \cdot SCI_{UB,\log 10} = 5.303 \cdot SCI_{UB,\log 10}$$

where $SCI_{UB,\log 10}$ is the SCI_{UB} value calculated using the equations listed above for the base-10 log transformed metrics.

Bioequivalence assessment of the clearance rate constant and flux

The k_{ij} and $J_{\text{in vivo},ij}$ metrics can exhibit both positive and negative values. Therefore, the bioequivalence analysis of either of these metrics, represented as $\bar{M}_{ij.}$, is performed as a pair-wise comparison as follows:

$\bar{H}_j = \bar{M}_{1j.} - \bar{M}_{2j.}$ = difference between formulations 1 and 2 in each subject *j*

$\bar{H} = \frac{1}{n}\sum_{j=1}^{n}\bar{H}_j$ = mean of the differences between formulations 1 and 2 averaged over *n* subjects

$s_H^2 = \frac{1}{n-1}\sum_{j=1}^{n}\left(H_j - \bar{H}\right)^2$ = variance of the differences between formulations 1 and 2 in *n* subjects

$\delta_{H,90\%} = \frac{s_H \cdot t_{0.95,n-1}}{\sqrt{n}}$ = projected half-width of the 90% confidence interval (CI) for the population mean difference

where $t_{0.95,n-1}$ is as defined for the traditional ABE assessment. The results are presented as the mean of the difference and the 90% CI: $\bar{H} \pm \delta_{H,90\%}$

The hypothesis that the mean of the differences is zero is calculated from the T-statistic, t_0, defined as

$$t_0 = \frac{\left|\left(\bar{H} - 0\right)\right|}{\left(s_H / \sqrt{n}\right)}$$

for *n*-1 degrees of freedom in a two-tailed test. Formulations were considered to be non-BE if the probability $p < 0.05$. Examples of the calculations comparing the US-C+ formulation to the US-Ref formulation for the k_{ij} and $J_{\text{in vivo},ij}$ metrics are presented in Table S3.

Table S1. ACV amounts (μg/cm^2) and log-transformed amounts recovered from the SC after uptake and clearance in the replicate samples (Q_{ijk} , replicate *k* of formulation *i* in subject *j*) from each of the 10 subjects for the US-C+ and US-Ref formulations (designated as formulations 1 and 2, respectively)

Subject Number	**US-C+ (*i* = 1) Uptake**						**US-C+ (*i* = 1) Clearance**					
	Q_{ij1}	Q_{ij2}	$\ln(Q_{ij1})$	$\ln(Q_{ij2})$	$\ln(\bar{Q}_{ij.})$	$\bar{Q}_{ij.}$	Q_{ij1}	Q_{ij2}	$\ln(Q_{ij1})$	$\ln(Q_{ij2})$	$\ln(\bar{Q}_{ij.})$	$\bar{Q}_{ij.}$
1	0.714	0.636	-0.337	-0.453	-0.395	0.674	0.434	0.378	-0.835	-0.973	-0.904	0.405
2	1.422	0.960	0.352	-0.041	0.156	1.168	1.472	0.822	0.387	-0.196	0.095	1.100
3	0.316	0.270	-1.152	-1.309	-1.231	0.292	0.290	0.686	-1.238	-0.377	-0.807	0.446
4	0.408	0.458	-0.896	-0.781	-0.839	0.432	0.276	0.384	-1.287	-0.957	-1.122	0.326
5	0.850	1.046	-0.163	0.045	-0.059	0.943	0.174	0.436	-1.749	-0.830	-1.289	0.275
6	1.040	0.530	0.039	-0.635	-0.298	0.742	0.558	0.150	-0.583	-1.897	-1.240	0.289
7	0.376	0.458	-0.978	-0.781	-0.880	0.415	0.330	0.322	-1.109	-1.133	-1.121	0.326
8	0.886	0.536	-0.121	-0.624	-0.372	0.689	0.590	0.836	-0.528	-0.179	-0.353	0.702
9	0.244	0.602	-1.411	-0.507	-0.959	0.383	0.170	0.290	-1.772	-1.238	-1.505	0.222
10	0.928	0.560	-0.075	-0.580	-0.327	0.721	0.656	0.712	-0.422	-0.340	-0.381	0.683
Mean, $\ln(\bar{Q}_{i..})$					-0.520						-0.863	
Std Dev					0.437						0.504	
90% CI					0.254						0.292	
Lower CI					-0.774						-1.155	
Upper CI					-0.267						-0.571	
Anti-log												
Mean, $\bar{Q}_{i..}$					0.594						0.422	
Lower CI					0.461						0.315	
Upper CI					0.765						0.565	

Table S1 (continued). ACV amounts (μg/cm^2) and log-transformed amounts recovered from the SC after uptake and clearance in the replicate samples (Q_{ijk} ,replicate *k* of formulation *i* in subject *j*) from each of the 10 subjects for the US-C+ and US-Ref formulations (designated as formulations 1 and 2, respectively).

**Subject Number**	**US-Ref (*i* = 2) Uptake**						**US-Ref (*i* = 2) Clearance**					
	Q_{ij1}	Q_{ij2}	$\ln(Q_{ij1})$	$\ln(Q_{ij2})$	$\ln(\bar{Q}_{ij.})$	$\bar{Q}_{ij.}$	Q_{ij1}	Q_{ij2}	$\ln(Q_{ij1})$	$\ln(Q_{ij2})$	$\ln(\bar{Q}_{ij.})$	$\bar{Q}_{ij.}$
1	0.904	0.512	-0.101	-0.669	-0.385	0.680	1.074	0.288	0.071	-1.245	-0.587	0.556
2	1.154	2.794	0.143	1.027	0.585	1.796	0.806	1.850	-0.216	0.615	0.200	1.221
3	0.364	0.296	-1.011	-1.217	-1.114	0.328	0.324	0.274	-1.127	-1.295	-1.211	0.298
4	0.668	0.442	-0.403	-0.816	-0.610	0.543	0.156	0.364	-1.858	-1.011	-1.434	0.238
5	0.818	3.344	-0.201	1.207	0.503	1.654	0.232	1.152	-1.461	0.141	-0.660	0.517
6	0.770	0.562	-0.261	-0.576	-0.419	0.658	0.642	0.346	-0.443	-1.061	-0.752	0.471
7	0.662	0.516	-0.412	-0.662	-0.537	0.584	0.402	0.366	-0.911	-1.005	-0.958	0.384
8	0.468	1.454	-0.759	0.374	-0.192	0.825	0.378	0.612	-0.973	-0.491	-0.732	0.481
9	0.222	0.504	-1.505	-0.685	-1.095	0.334	0.098	0.696	-2.323	-0.362	-1.343	0.261
10	1.152	0.306	0.141	-1.184	-0.521	0.594	0.474	0.664	-0.747	-0.409	-0.578	0.561
Mean, $\ln(\bar{Q}_{i..})$					-0.379						-0.805	
Std Dev					0.567						0.473	
90% CI					0.329						0.274	
Lower CI					-0.707						-1.080	
Upper CI					-0.050						-0.531	
Anti-log												
Mean, $\bar{Q}_{i..}$					0.685						0.447	
Lower CI					0.493						0.340	
Upper CI					0.952						0.588	

Table S2. Calculated values for the drug flux from the SC into the underlying viable tissue during the clearance period ($J_{in\ vivo}$) and the first-order SC clearance rate constant (k) from each of the 10 subjects for the US-C+ and US-Ref formulations (designated as formulations 1 and 2, respectively).

Subject Number	$J_{in\ vivo,ij}$ (ng cm^{-2} h^{-1})		$100 \cdot k_{ij}$ (1/h)	
	US-C+ (i = 1)	US-Ref (i = 2)	US-C+ (i = 1)	US-Ref (i = 2)
1	15.81	7.30	2.995	1.185
2	4.02	33.80	0.355	2.268
3	-9.05	1.78	-2.490	0.570
4	6.28	17.95	1.668	4.849
5	39.26	66.88	7.239	6.841
6	26.65	10.97	5.544	1.961
7	5.24	11.82	1.420	2.477
8	-0.78	20.23	-0.111	3.173
9	9.48	4.31	3.211	1.456
10	2.20	1.92	0.314	0.333
Mean	9.91	17.70	2.014	2.511
Std Dev	14.06	19.87	2.849	2.011
90% CI	8.15	11.52	1.652	1.166

Table S3. Bioequivalence evaluation of the US-C+ and US-Ref formulations (designated as formulations 1 and 2, respectively) calculated as the difference between the formulations of the log transformed ACV amount in the tape strips ($\overline{I}_j$) collected after uptake and clearance, and calculated as the difference between the formulations of the flux ($J_{\text{in vivo}}$) and the first-order clearance rate constant (k).

Subject Number	$\overline{I}_j = \overline{Z}_{1j.} - \overline{Z}_{2j.}$ (µg /cm^2)	
	Uptake	Clearance
1	-0.0095	-0.31708
2	-0.4297	-0.10445
3	-0.1167	0.40344
4	-0.2287	0.31202
5	-0.5619	-0.62965
6	0.1210	-0.48802
7	-0.3425	-0.16272
8	-0.1798	0.37856
9	0.1361	-0.16232
10	0.1941	0.19737
$\overline{I}$	-0.1418	-0.05728
s_I	0.2551	0.36643
$\delta_{I,90\%}$	0.1479	0.21242
$\overline{I} - \delta_{I,90\%}$	-0.2897	-0.26970
$\overline{I} + \delta_{I,90\%}$	0.0061	0.155
GMR	0.867	0.944
$GMR_{90\%,lower}$	0.749	0.764
$GMR_{90\%,upper}$	1.006	1.168

Subject Number	$\overline{H}_j = \overline{M}_{1j.} - \overline{M}_{2j.}$	
	$J_{\text{in vivo},1j} - J_{\text{in vivo},2j}$ (ng cm^{-2} h^{-1})	$100 \cdot \left(k_{2j} - k_{1j}\right)$ (1/h)
1	8.51	1.81
2	-29.77	-1.910
3	-10.84	-3.06
4	-11.67	-3.18
5	-27.61	0.40
6	15.68	3.58
7	-6.58	-1.06
8	-21.01	-3.28
9	5.17	1.76
10	0.28	-0.02
$\overline{H}$	-7.78	-0.50
s_H	15.37	2.40
$\delta_{H,90\%}$	8.91	1.39
p ($\overline{H} = 0$)	0.144	0.529

Table S4. Calculation of the within subject variance (s_{W2}^2) for the US-Ref formulation (designated as formulation 2).

Subject Number	$\left[Z_{2jk} - \bar{Z}_{2j.}\right]^2$			
	Uptake		Clearance	
	k = 1	k = 2	k = 1	k = 2
1	0.0808	0.0808	0.4331	0.4331
2	0.1955	0.1955	0.1726	0.1726
3	0.0107	0.0107	0.0070	0.0070
4	0.0426	0.0426	0.1795	0.1795
5	0.4957	0.4957	0.6420	0.6420
6	0.0248	0.0248	0.0955	0.0955
7	0.0155	0.0155	0.0022	0.0022
8	0.3213	0.3213	0.0580	0.0580
9	0.1681	0.1681	0.9608	0.9608
10	0.4394	0.4394	0.0284	0.0284
**Sum**	3.5885		5.1583	
s_{W2}^2	0.3588		0.5158	
s_{W2}	0.5990		0.7182	

Table S5. Scaled average bioequivalence (SABE) calculations comparing the US-C+ and US-Ref formulations (designated as formulations 1 and 2, respectively) for the log transformed ACV amount in the tape strips collected after uptake and clearance for *m* = 1.25.

Input Parameters						
σ_{W0}	m	θ	n	nr	$t_{0.95,(n-1)}$	$\chi^2_{0.95,nr(n-1)}$
0.25	1.25	0.7967	10	2	1.8331	18.3070

Calculated Values												
	$\bar{I}$	s_I^2	s_{W2}	X	Y	X'_β	Y'_β	$X'_\beta - X$	$Y'_\beta - Y$	V	SCI_{UB}	GMR
Uptake	-0.1418	0.0651	0.5990	0.0136	-0.2859	0.0839	-0.1562	0.0703	0.1297	0.0218	-0.1247	0.867
Clearance	-0.0573	0.1343	0.7182	-0.0101	-0.4110	0.0727	-0.2245	0.0829	0.1865	0.0416	-0.2170	0.944

Table S6. Within subject standard deviation of each product (s_W) and the standard deviation of the compared products (s_I) evaluated from the natural log transformed drug mass in the SC measured in each study. Values used in the power simulation studies shown in Figures S5-S7 are designated.

s_W	Study 1			Study 2		
	US-Ref [a,b]	US-C+	UK-Test	AT-Ref [a,b]	AT-C+	US-Test
Uptake	0.599	0.321	0.840	0.695	0.435	0.463
Clearance	0.718	0.458	0.794	0.426	0.389	0.317
s_I	Study 1			Study 2		
	US-C+/ US-Ref [a]	UK-Test/ US-Ref [b]	UK-Test /US-C+	AT-C+/ AT-Ref [a]	AT-Ref/ US-Test [b]	AT-C+/ US-Test
Uptake	0.255	0.296	0.320	0.379	0.469	0.545
Clearance	0.366	0.484	0.625	0.381	0.419	0.333

[a] Values of s_W and s_I used in the power simulations for comparisons of the positive control to the corresponding reference product in Studies 1 and 2 for uptake and clearance shown in Figures S5 and S8.

[b] Values of s_W and s_I used in the power simulations for comparisons of the test to the reference product in Studies 1 and 2 for uptake and clearance shown in Figures S6, S7 and S9.

Power simulations of bioequivalence assessments

We evaluated the number of subjects required to adequately power the traditional ABE and SABE methods for the *m* = 1.25 limit by performing simulations. For each such study, the inputs of the power function are the within-subject standard deviation of the reference product (s_{W2} in an assessment of the difference between formulations 1 and 2), the between-subjects standard deviation (s_I), the number of subjects and the number of replicates. This process is repeated 500,000 times under the assumption of bioequivalence. The value of the power is then the percentage of these trials that correctly captured the equivalence relationship between the two products. Table S6 lists the within-subject standard deviation (s_W) for all products and s_I for all product pairs evaluated for bioequivalence in this study; values used in the power simulation calculations are designated.

The power simulation results for *m* = 1.25 are presented in Figure S5 for comparisons of the positive control to the corresponding reference product (i.e., US-C+ to US-Ref and AT-C+ to AT-Ref in Studies 1 and 2, respectively), and in Figures S6 and S7 for comparisons of the test to reference product (i.e., UK-Test to US-Ref and US-Test to AT-Ref in Studies 1 and 2, respectively). The expected minimum number of subjects required to achieve a statistical power of at least 80% is listed in Table S7. The SABE methodology was estimated to achieve a statistical power close to 80% with 10 subjects for the products compared in Study 1 for both uptake and clearance, and for uptake in Study 2 (which involved a different cohort of 10 subjects); see Figures S5 and S6 and Table S7. Approximately 15 subjects are needed to adequately power the clearance results in Study 2 (Figure S6). By comparison, the traditional ABE methodology is estimated to require, between 15 and 50 subjects to achieve the same power, with fewer subjects needed in the assessment of the positive control with the corresponding reference product (Figures S5 and S6 and Table S7). Increasing replication from two to three sites for each product in this study had minimal benefit, reducing the number of subjects required to achieve the same power in the SABE assessment by approximately one subject (Figure S7).

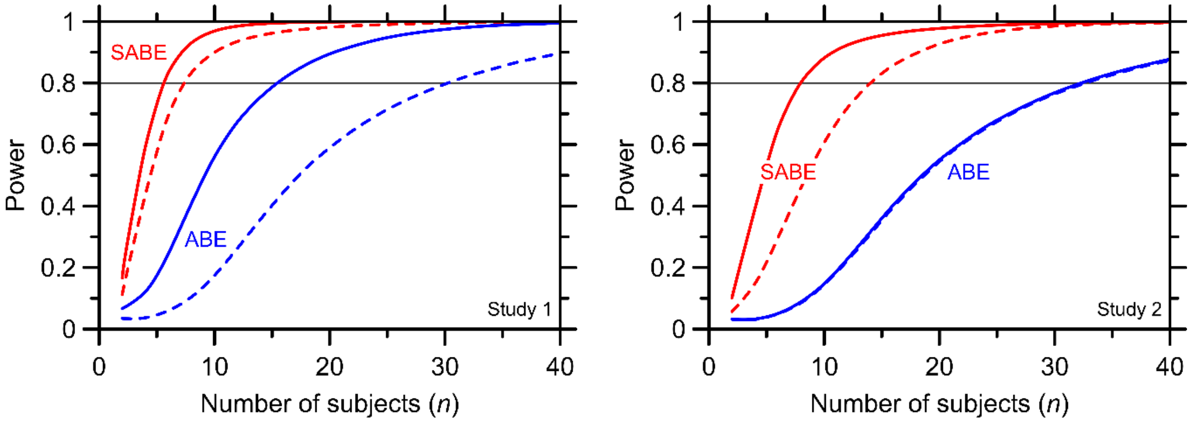

Figure S5. Estimated power as a function of the number of subjects (*n*) for traditional average bioequivalence (ABE) and scaled average bioequivalence (SABE) assessments of the positive control to the corresponding reference product in Studies 1 and 2 for the bioequivalence margin *m* = 1.25: uptake (solid) and clearance (dashed). The statistical power of 0.8 commonly recommended for acceptance by regulatory agencies is indicated.

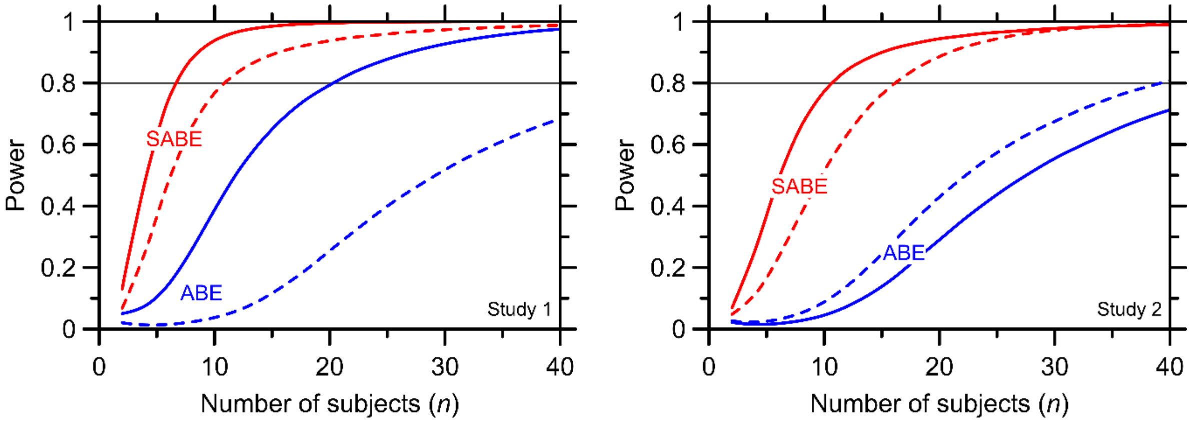

Figure S6. Estimated power as a function of the number of subjects (*n*) for traditional average bioequivalence (ABE) and scaled average bioequivalence (SABE) assessments of the test to reference products in Studies 1 and 2 for the bioequivalence margin *m* = 1.25: uptake (solid) and clearance (dashed). The statistical power of 0.8 commonly recommended for acceptance by regulatory agencies is indicated.

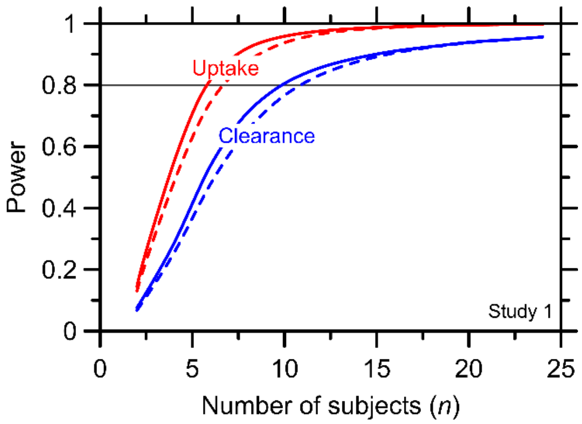

Figure S7. Estimated power as a function of the number of subject (*n*) when the number of replicates for comparing the test and reference products in Studies 1 and 2 is increased from two (dashed) to three (solid) for the bioequivalence margin *m* = 1.25. The statistical power of 0.8 commonly recommended for acceptance by regulatory agencies is indicated.

Table S7. Estimated minimum number of subjects required to achieve a statistical power power of at least 80% for the bioequivalence margin (*m*) equal to 1.25 and 1.33

	ABE				SABE			
Study 1	US-C+/US-Ref		UK-Test/US-Ref		US-C+/US-Ref		UK-Test/US-Ref	
m	1.25	1.33	1.25	1.33	1.25	1.33	1.25	1.33
Uptake	16	10	21	12	6	5	7	6
Clearance	31	18	>40	29	8	6	11	8
	ABE				SABE			
Study 2	AT-C+/AT-Ref		US-Test/AT-Ref		AT-C+/AT-Ref		US-Test/AT-Ref	
m	1.25	1.33	1.25	1.33	1.25	1.33	1.25	1.33
Uptake	33	19	>40	28	8	6	11	8
Clearance	33	19	40	23	15	10	17	11

The power simulation results for *m* = 1.25 compared with *m* = 1.33 are presented in Figure S8 for assessments of the positive control to the corresponding reference product (i.e., US-C+ to US-Ref and AT-C+ to AT-Ref in Studies 1 and 2, respectively), and in Figure S9 for assessments of the test to reference product (i.e., UK-Test to US-Ref and US-Test to AT-Ref in Studies 1 and 2, respectively).

Table S7 lists the expected minimum number of subjects required to achieve a statistical power of at least 80% for *m* equal to both 1.25 and 1.33.

The power of a bioequivalence study using cutaneous pharmacokinetic endpoints can be substantially increased by widening the bioequivalence limits from the traditional *m* = 1.25 to *m* = 1.33 for an ABE assessment. The advantage of this approach is that fewer subjects are needed to power the study. However, the disadvantage of widening the bioequivalence limits is that it essentially lowers the standard for comparability of the test and reference products. Using an SABE analysis instead of an ABE analysis, while maintaining the traditional bioequivalence limit of *m* = 1.25, increases the power of the study to an even greater degree than by widening the bioequivalence limits for an ABE analysis to *m* = 1.33. The additional power gained by widening the bioequivalence limits from *m* = 1.25 to *m* = 1.33 in an SABE analysis is much smaller than for the ABE assessment. The results presented in Figure S8 confirm that the comparison of AT-C+ and AT-Ref products in Study 2 with 10 subjects was slightly underpowered for an SABE assessment at *m* = 1.25, but is adequately powered at *m* = 1.33 (in which case the SABE assessment successfully demonstrated bioequivalence as shown in Table 4 in the paper).

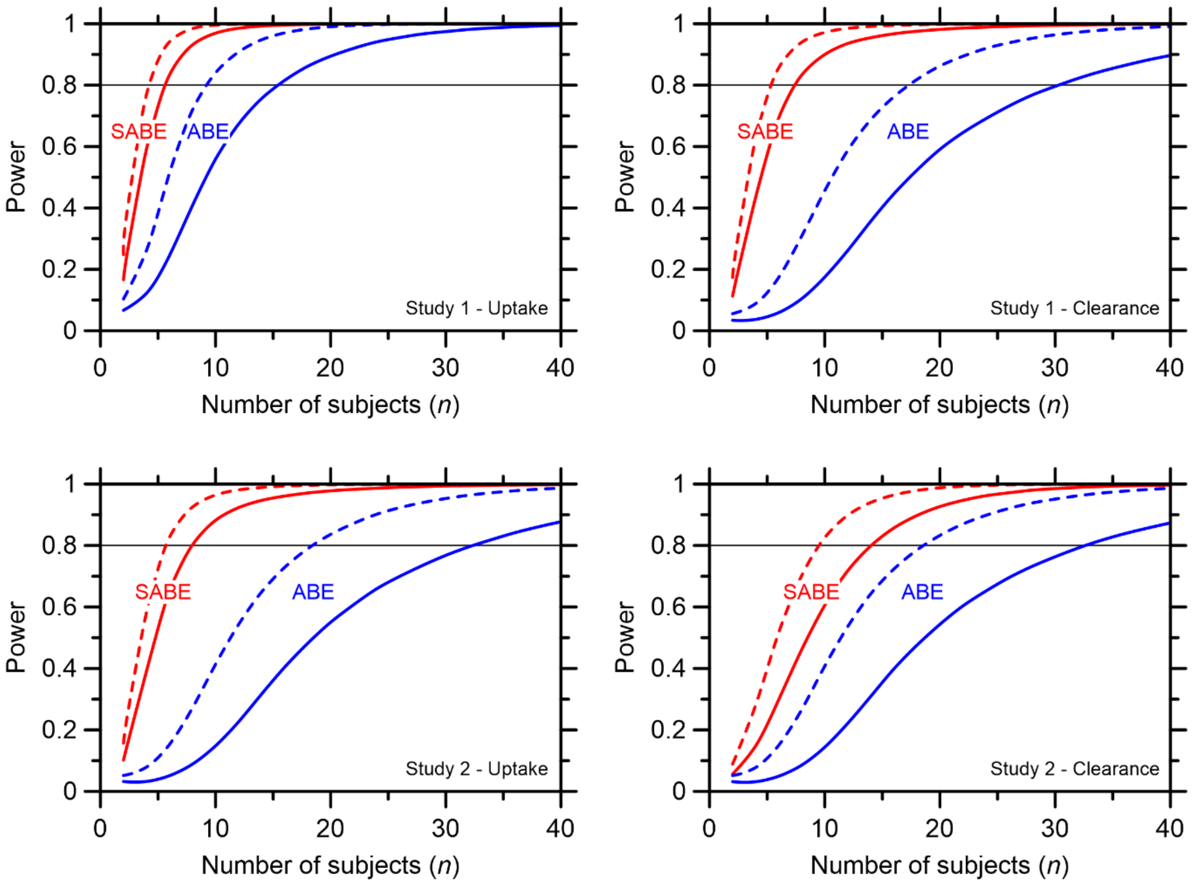

Figure S8. Estimated power as a function of the number of subjects (*n*) for traditional average bioequivalence (ABE) and scaled average bioequivalence (SABE) assessments of the positive control to the corresponding reference product in Studies 1 (US-C+ versus US-Ref) and 2 (AT-C+ versus AT-Ref) after uptake (left) and clearance (right) for the bioequivalence margin *m*= 1.25 (solid) and 1.33 (dashed). The statistical power of 0.8 commonly recommended for acceptance by regulatory agencies is indicated.

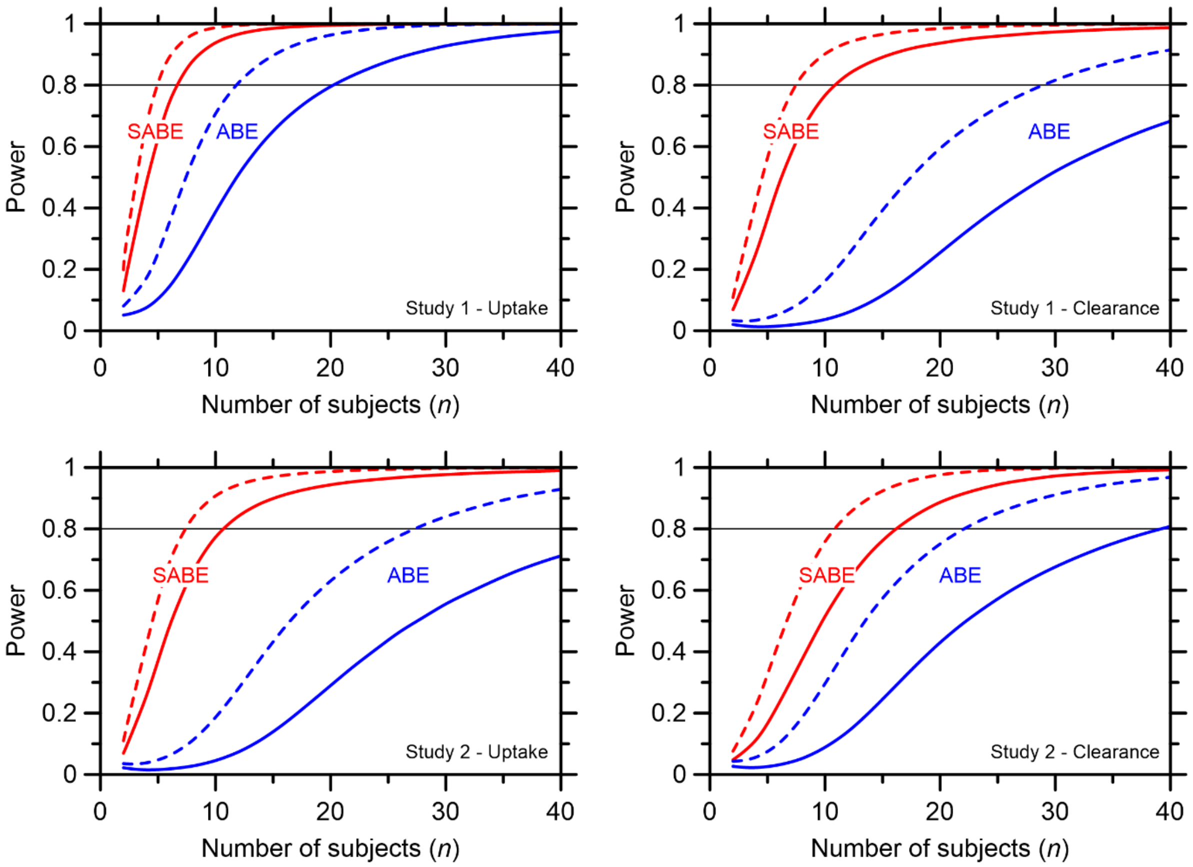

Figure S9. Estimated power as a function of the number of subjects (*n*) for traditional average bioequivalence (ABE) and scaled average bioequivalence (SABE) assessments of the test to reference product in Studies 1 (UK-Test versus US-Ref) and 2 (US-Test versus AT-Ref) after uptake (left) and clearance (right) for the bioequivalence margin *m*= 1.25 (solid) and 1.33 (dashed). The statistical power of 0.8 commonly recommended for acceptance by regulatory agencies is indicated.

References

1. N'Dri-Stempfer B, Navidi WC, Guy RH, Bunge AL. Improved bioequivalence assessment of topical dermatological drug products using dermatopharmacokinetics. Pharm Res 2009;26(2):316-328.

2. Cordery SF, Pensado A, Chiu WS, Shehab MZ, Bunge AL, Delgado-Charro MB, Guy RH. Topical bioavailability of diclofenac from locally-acting, dermatological formulations. Int J Pharm 2017;529(1–2):55-64.

3. Rantou E, Schuirmann D, Raney S., Grosser, S. A statistical approach for establishing bioequivalence in topical dermatological drug products. Pharm Res 2019; this issue.

4. Schuirmann DJ. A comparison of the two one-sided tests procedure and the power approach for assessing the equivalence of average bioavailability. J Pharmacokinet Biopharm. 1987;15, 657-680.

5. Shin S H, Rantou E, Raney SG, Ghosh P, Hassan H, Stinchcomb A. Cutaneous pharmacokinetics of acyclovir cream 5% products: Evaluating bioequivalence with an *in vitro* permeation test and an adaptation of scaled average bioequivalence. Pharm Res 2019; this issue
